# Types of Bone Substitutes and Their Application in Regenerative Medicine: A Systematic Review

**DOI:** 10.3390/jfb16090341

**Published:** 2025-09-09

**Authors:** Nikoleta Ivanova, Stoyan Ivanov, Stefan Peev, Tsanka Dikova

**Affiliations:** 1 Department of Biology, Faculty of Pharmacy, Medical University of Varna, 9002 Varna, Bulgaria; 2 Department of Orthopedics and Traumatology, Faculty of Medicine, Medical University of Varna, 9002 Varna, Bulgaria; st.ivanov@mu-varna.bg; 3 Department of Periodontology and Dental Implantology, Faculty of Dental Medicine, Medical University of Varna, 9002 Varna, Bulgaria; stefan.peev@mu-varna.bg; 4 Department of Dental Material Science and Prosthetic Dental Medicine, Faculty of Dental Medicine, Medical University of Varna, 9002 Varna, Bulgaria

**Keywords:** bone tissue, stem cells, clinical efficacy, bone defects, remodelling

## Abstract

Background: The growing demand for effective methods of bone tissue regeneration highlights the relevance of studying modern bone substitutes and their applications in regenerative medicine. The aim of this work was to conduct a comprehensive analysis of the biological, mechanical, and clinical characteristics of various types of bone substitutes to determine their potential in regenerative medicine. Methods: The study was performed as a systematic literature review in accordance with PRISMA guidelines, analyzing 68 high-quality scientific sources from 2019 to May 2025, using the PubMed, Scopus, Web of Science, and Google Scholar databases. Results: It was established that autogenous grafts exhibit the highest osteogenic properties due to the presence of growth factors BMP-2, BMP-7, and concentrated growth factors; however, their use is limited by donor site morbidity in 20–30% of patients and the requirement to treat 6% of fractures complicated by non-union. Allogeneic and xenogeneic substitutes provide structural support for large defects but require intensive processing in accordance with European Directives 2004/23/EC and 2006/86/EC to minimize the risk of infection transmission. Synthetic substitutes based on calcium phosphate ceramics with pore sizes ranging from 23 to 210 micrometres demonstrate excellent biocompatibility and controlled degradation, with β-tricalcium phosphate exhibiting optimal characteristics for long-term applications compared to calcium sulphate. Conclusions: The findings of the study highlight the necessity of a personalized approach in selecting bone substitutes, considering the specific requirements of medical specialities, and support the development of hybrid biomaterials to combine structural strength with biological activity.

## 1. Introduction

The field of regenerative medicine increasingly requires effective bone regeneration methods, particularly for orthopedic, maxillofacial, and dental procedures. Bone defects resulting from trauma, disease, or surgery have been identified as a significant clinical problem. While minor defects may heal spontaneously, more serious defects necessitate treatment through transplantation or the use of bone substitutes. Traditional transplantation methods, as noted by M.P. Ferraz, include autografts, allografts, and xenografts, which remain routinely used but are associated with complications such as donor site morbidity, immune rejection, and variable integration rates [1]. In response, synthetic and bioengineered bone substitutes have become a central focus in regenerative medicine.

Despite significant advances in tissue engineering and biomaterials, no single, universally accepted optimal bone substitute exists. This is due to the inability to combine, within a single material, all the necessary properties: the biological activity of autografts, the unlimited availability of synthetic materials, controlled degradation, the absence of immune reactions, and adaptability to diverse clinical requirements—from high mechanical load-bearing to rapid resorption. According to M. Heitzer et al., the applicability of these substitutes is determined by their osteoconductivity, osteoinductivity, and mechanical strength, and most importantly, their biocompatibility [2]. Scaffolds offer considerable potential when combined with stem cells, bioactive coatings, and 3D bioprinting; however, challenges remain in optimizing resorption rates, mechanical properties, and clinical relevance.

Overall, H. Fattahian et al. in their review, described the main types of bone substitutes, along with their advantages and limitations [3]. They emphasized that traditional methods such as autografts and allografts possess several shortcomings, thereby prompting the active development of tissue engineering approaches that integrate biomaterials, growth factors, stem cells, and even 3D printing technologies to create ideal bone substitutes. The work of U. Dahiya et al. highlighted the limitations of autologous grafts and examined synthetic substitutes, bioceramics, and polymers with osteoconductive, osteoinductive, and biocompatible properties as promising materials for bone regeneration [4].

In turn, B. Manzini et al. emphasized the importance of biofactors and stem cells in the regeneration process, highlighting the need for further research to develop biomimetic bone substitutes capable of effectively interacting with the body and supporting tissue regeneration [5]. Research represented by the work of M. Miletić et al. which examined the combination of artificial β-tricalcium phosphate with periodontal ligament mesenchymal stem cells, noting a significant increase in regenerative potential compared to cell-free material [6]. This underscores the importance of combined approaches involving cells and substitutes.

Another study by S. Mukhlis et al. highlights the role of pericytes—cells that promote angiogenesis and vascular regeneration in bone tissue [7]. The authors emphasize the potential of using these cells in conjunction with biomaterials to enhance regenerative outcomes, while also noting the need for further investigation into their mechanisms of action and optimal treatment regimens. Other studies, including those by F. Dőri et al. have assessed the combination of biomaterials with various methods for stimulating tissue regeneration, such as the use of plasma, which enhances the integration of membranes and bone substitutes [8]. However, they acknowledge that the correlation between histological changes and clinical outcomes still requires further clarification. At the same time, Q. Zhang addressed the issue of bone cell ageing and osteoporosis, which limit bone tissue regeneration [9]. This study considered the potential use of substitutes containing therapeutic agents to simultaneously address these pathologies, thereby emphasizing the complex and multifactorial nature of the problem.

Overall, current research indicates that successful bone regeneration requires an integrated approach combining innovative biomaterials, cell technologies, growth factors, and vascular growth stimulation. However, many gaps remain, particularly in the optimization of material–cell combinations, the understanding of implant–immune system interactions, and the standardization of clinical protocols to ensure long-term success. Therefore, the aim of this study was to conduct a systematic review of the use of bone substitutes in regenerative medicine, based on an analysis of scientific publications from 2019 to 2025 in accordance with the PRISMA methodology. To achieve this aim, the following research tasks were undertaken: to conduct a comparative analysis of the biological and clinical characteristics of different groups of bone substitutes (autogenous, allogeneic, xenogeneic, and synthetic), with an assessment of their osteogenic, osteoinductive, and osteoconductive potential; to evaluate the clinical and preclinical effectiveness of bioengineered and synthetic bone graft substitutes, with particular attention to their use in bone tissue regeneration and integration into host tissues; and to identify promising directions for development and current challenges in bone substitute technologies, including their limitations and areas requiring further research.

## 2. Materials and Methods

This study is a systematic review of the literature, conducted in accordance with the PRISMA (Preferred Reporting Items for Systematic Reviews and Meta-Analyses) guidelines to ensure methodological rigour and transparency, registered in PROSPERO under registration number CRD420251117103. The time frame of the study encompassed publications from January 2019 to May 2025, allowing for the analysis of recent advances in the field of bone substitutes and their application in regenerative medicine. As this is a systematic review of published scientific sources and does not involve clinical interventions, ethical approval from the institutional ethics committee was not required.

Data collection was performed through a comprehensive search of the electronic scientometric databases PubMed, Scopus, Web of Science, and Google Scholar. The first stage of the search strategy identified 312 publications in PubMed, 287 sources in Scopus, 254 articles in Web of Science, and 198 additional sources in Google Scholar, resulting in a total of 1051 publications. A combination of keywords and Medical Subject Headings (MeSH) terms was used to ensure maximum coverage of relevant literature, including: “bone substitutes” OR “bone grafts” AND “regenerative medicine,” “autogenous bone grafts” OR “autologous bone transplants,” “allogeneic bone substitutes” OR “allografts,” “xenogeneic bone substitutes” OR “xenografts,” “synthetic bone substitutes” OR “bioceramics” OR “calcium phosphate ceramics,” “osteoconductivity” OR “osteoinductivity” OR “osteogenesis,” “bone tissue engineering” AND “scaffold materials.” Search queries were formulated using Boolean operators to optimize search results and exclude irrelevant publications.

Inclusion criteria comprised original studies, systematic reviews, and meta-analyses published in peer-reviewed journals in English within the specified period. The analysis included papers addressing the biological properties, mechanical characteristics, fabrication methods, and clinical applications of autogenous, allogeneic, xenogeneic, and synthetic bone substitutes. Particular attention was paid to studies evaluating the osteoinductive, osteoconductive, and osteogenic properties of materials, as well as their biocompatibility and degradation rate both in vivo and in vitro. The exclusion criteria included publications dated before 2019 (excluding European Directives and documents from the European Medicines Agency), articles in languages other than English, editorial articles, commentaries, and letters to the editor. Studies not directly addressing bone substitutes or their applications in regenerative medicine, papers with insufficient methodological quality, and publications without access to the full text were also excluded. The methodological quality of the included studies was assessed based on the following criteria: a clear description of the methodology, adequate sample size, use of appropriate control groups, statistical analysis of results, and transparency in data presentation. For clinical studies, additional consideration was given to the level of evidence according to the hierarchy of medical evidence and the presence of randomisation. The selection and analysis of publications were conducted by two independent reviewers, with subsequent discussion of discrepancies to reach a consensus. In cases of disagreement, a third expert was consulted for the final decision.

Systematization and management of bibliographic data were carried out using specialized software: Mendeley Desktop (version 1.19.8) and Zotero (version 6.0.30), which enabled effective organization of the selection and categorisation process for publications. Duplicate records were identified and removed using Mendeley’s built-in deduplication algorithm, followed by manual verification. Bibliographic data were exported from databases in RIS and BibTeX formats to ensure compatibility with reference management programmes. Full-text analysis was performed using Adobe Acrobat Reader DC (version 2024.001.20643), employing annotation and highlighting functions to mark key text fragments. Microsoft Excel 365 (version 16.0.17328.20162) was used to construct a data matrix and conduct preliminary analysis, systematizing information on study type, year of publication, type of bone substitute, research methodology, and main results. Visualization of the literature selection process, following PRISMA methodology, was performed using Microsoft Visio 2021 (version 16.0.14332.20447).

**Figure 1:**
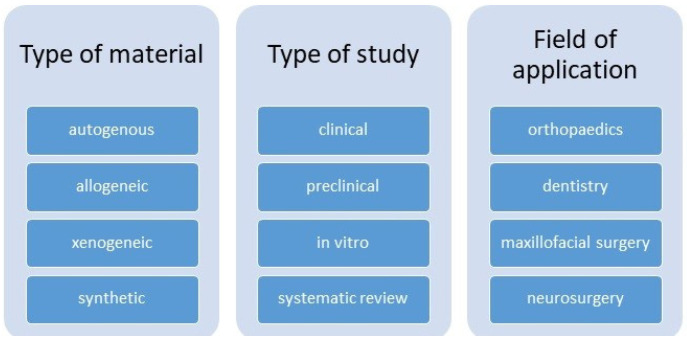
Type of material, type of study, and field of application of bone substitutes included in the review article.

The literature review is organized according to the classifications presented in Figure 1. 

The analysis is conducted across four primary categories of bone substitutes—autogenous, allogeneic, xenogeneic, and synthetic—assessing their biological properties, mechanical characteristics, and clinical efficacy. The studies from which the bone substitute groups are analyzed are clinical, preclinical, in vitro, and systematic reviews. The fields of application in which they are observed are orthopedics, dentistry, maxillofacial surgery, and neurosurgery. In addition, the features of each material type are compared, their indications for use in various medical specialties are systematized, and key areas requiring further investigation are identified. The synthesis of data allows for the identification of major trends in the development of new materials, including the use of 3D printing, the incorporation of growth factors, and the application of stem cells to enhance regenerative potential.

## 3. Results and Discussion

**Figure 2:**
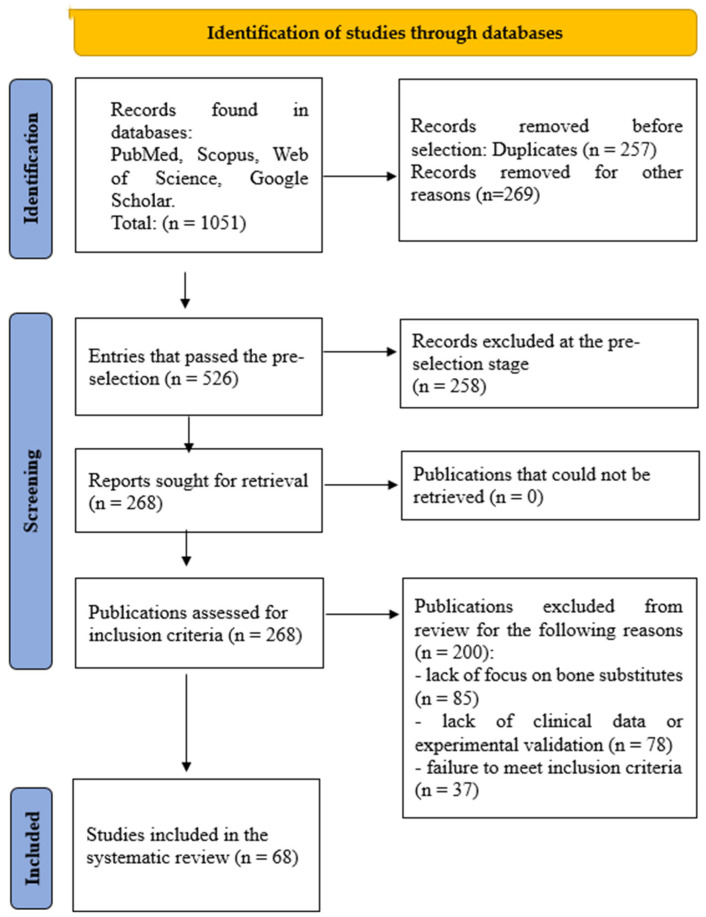
PRISMA flow diagram of the study selection process.

The selection process was conducted in three consecutive stages in accordance with the PRISMA methodology, as illustrated in the flowchart (Figure 2). As a result, 68 high-quality scientific sources were included in the final systematic analysis.

Table 1 presents the characteristics of the studies included in this systematic review.

**Figure 3:**
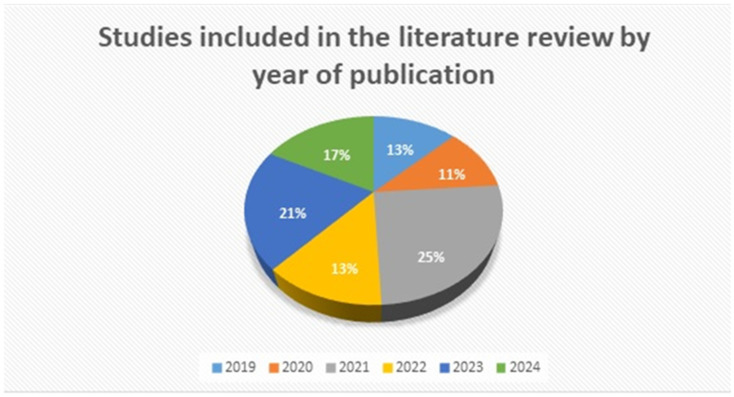
Annual distribution of studies included in the literature review (2019–2024).

Figure 3 shows the publications, included in this literature review, distributed by year of publication. A clear trend of high research interest in bone substitutes and regenerative medicine can be seen. Notably, the number of published studies increased significantly from 2021 onwards, with a peak in 2021 and a subsequent high publication rate in 2023 and 2024. This trend indicates that bone tissue engineering continues to be a dynamic and rapidly evolving field.

### 3.1. Importance of Bone Substitutes in Regenerative Medicine

Modern regenerative medicine employs bone substitutes as essential tools for the treatment of bone defects caused by trauma, tumours, congenital malformations, and degenerative diseases. The growing demand for effective bone regeneration has directed much research towards the development of biomaterials capable of replacing damaged bone tissue and supporting the body’s natural healing processes. A systematic review by M.P. Ferraz [1] outlines the widespread use of bone grafts in dental and orthopedic procedures, including the treatment of fractures, repair of non-union bone tissue, and reconstruction following tumour-related injuries. Evidence confirms that autografts remain the reference standard due to their superior osteogenic potential and biocompatibility. However, their use is limited by donor site morbidity and restricted availability, prompting the ongoing search for alternative bone substitutes.

Current scientific research identifies several key clinical scenarios necessitating the use of bone grafts. Trauma surgery is the leading indication for bone graft application. A clinical study by M. Rupp et al., which analyzed over one million surgical procedures over a ten-year period, demonstrated that bone substitutes are commonly employed to treat bone defects and delayed or non-union fractures—especially in complex cases where an insufficient quantity of bone tissue is available for successful healing [10]. Studies indicate that approximately 6% of all fractures are complicated by non-union, requiring additional surgical intervention using bone substitutes to stimulate the regenerative process. Thus, trauma surgery remains the largest consumer of bone substitutes, where they play a critical role in managing complex fractures that do not respond to standard treatment. The prevalence of fracture non-union highlights the need to develop more effective osteoinductive materials for this field.

Spinal surgery represents another key application area for bone substitutes. A systematic review by M. Laubach et al. reports their use in spinal fusion procedures and the stabilization of vertebral segments [11]. These procedures are essential in the management of degenerative spinal conditions, traumatic injuries, and spinal deformities. The particular demands of spinal surgery require bone substitutes to offer a unique combination of mechanical strength—to support the spine—and biological activity—to ensure reliable vertebral fusion. This makes spinal applications especially demanding in terms of material quality. The treatment of oncological bone lesions also necessitates the use of bone substitutes. A review by A. Santoro et al. highlights the need for bone reconstruction following tumour resection, to restore both the structural integrity of the skeletal system and the functional capacity of affected limbs [12]. In these cases, bone substitutes help preserve anatomical structure and mobility. Oncological surgery presents unique challenges for bone substitute materials, as it requires a balance between radical tumour resection and the preservation of function—necessitating materials with reliable biointegration and the capacity to fill extensive bone defects.

Human bone grafting techniques developed in the past have been successfully refined, leading to the creation of modern synthetic materials that offer improved stability and biocompatibility. Autogenous bone grafts have been referenced in medical literature since the 19th century and have been employed by surgeons to reconstruct bones using material harvested from the fibula or iliac crest. A preclinical study by P. Tournier et al. discussed the historical development of bone grafting techniques and the gradual shift towards bone substitutes [13]. The authors traced the introduction of allografts in the mid-20th century, when they became widely accepted due to their affordability and the reduced surgical burden placed on the patient. However, concerns regarding immune rejection and disease transmission have driven the development of safer alternatives. The study suggested that future advancements in bioprinting, nanotechnology, and tissue engineering will be essential for creating the next generation of bone substitutes, given the currently unpredictable clinical behaviour of these materials.

The ethical considerations associated with the use of various bone graft types, including allogeneic and xenogeneic materials, are complex and multifaceted. As noted in a case study by S. Parikh et al., the use of allogeneic bone grafts raises significant ethical questions, particularly regarding informed consent from donors—especially when the donor is a minor who may not fully comprehend the implications of the procedure [14]. In such cases, decisions are made by parents or legal representatives, which introduces ethical dilemmas surrounding voluntariness and the protection of the donor’s interests. This study also underlined the importance of strict material safety monitoring, the prevention of infection transmission, and the maintenance of anonymity, all of which are critical to ethical transplantation practices.

A systematic review by M. Sharifi et al. pointed out that xenografts derived from animals present additional ethical concerns related to animal welfare and humane treatment [15]. The authors emphasized the necessity of accounting for cultural and religious considerations, as some patients may regard the use of animal-derived tissues as unacceptable. In such cases, it is essential to respect patients’ beliefs and to offer alternative treatment options.

According to I. Ana, in a chapter of the monograph dedicated to bone substitutes in dental implantology, an ethical approach to the use of bone grafts in clinical practice should also encompass procedural transparency, equitable access to materials, and the avoidance of commercialisation, which may lead to conflicts of interest [16]. The author emphasizes that maintaining a balance between medical efficacy and ethical standards is crucial for the successful and responsible implementation of transplantation procedures. The ethical dimensions of bone substitute use add an additional layer of complexity to the selection of optimal materials, requiring consideration not only of clinical effectiveness but also of cultural, religious, and legal factors. This underscores the need for the development of synthetic alternatives that circumvent the ethical dilemmas associated with donor-derived tissues.

Thus, based on the analysis of the aforementioned studies, it can be concluded that ethical considerations in the use of allogeneic and xenogeneic bone grafts are multifaceted and necessitate a comprehensive approach that includes the protection of donor rights, patient safety, respect for cultural values, and transparency in clinical practice. Bone substitutes have become an essential component of modern regenerative medicine, offering solutions to critical clinical challenges ranging from trauma reconstruction to oncological surgery. Their significance continues to grow, particularly in the context of global population ageing, the rising incidence of osteoporotic fractures, and the expanding indications for complex reconstructive procedures. At the same time, despite the rapid advancement of biomaterials, none of the currently available substitutes fully replicate the biofunctional properties of natural bone tissue, as evidenced by limitations in osteogenic, osteoinductive, and osteoconductive performance. A major challenge remains the absence of universal protocols for material selection tailored to specific clinical scenarios, along with limited long-term clinical data on the efficacy of different types of substitutes. Given the imperative to shorten rehabilitation periods, reduce complication risks, and improve functional outcomes, systematic analysis of the properties and effectiveness of bone substitutes—as well as the development of personalized selection strategies—represents one of the key directions for future advancement in regenerative medicine.

### 3.2. Main Categories and Characteristics of Modern Bone Substitutes

#### 3.2.1. Autologous Bone Grafts

Autologous bone grafts are employed in patients for bone regeneration owing to their highly effective biological characteristics, particularly their osteogenic potential, osteoinductivity, and osteoconductivity. In autologous graft procedures, surgeons harvest the patient’s own tissue from four primary donor sites: the iliac crest, fibula, tibia, and mandibular symphysis. A review article by O.S. Janjua et al. analyzed the structure and cellular behaviour of autologous grafts, highlighting the viability of their osteoblasts and the presence of endogenous growth factors that promote bone formation [17]. The study emphasized the natural process of bone remodelling facilitated by living bone cells and bone morphogenetic proteins (BMPs) in such grafts, which is significantly superior to that observed in allografts and synthetic substitutes. However, the review also acknowledged limitations such as the finite quantity of donor bone and the necessity to identify substitutes in cases requiring multiple transplantation procedures.

The effectiveness of autografts in bone tissue regeneration is primarily attributed to the presence of specific growth factors and proteins that play a crucial role in osteogenesis, angiogenesis, and bone remodelling. These biomolecules initiate cellular signalling cascades that activate the proliferation, differentiation, and functional activity of osteoblasts, regulate extracellular matrix formation, and maintain tissue homeostasis. As reported in a randomized clinical trial by S. Malik et al., one of the key contributors to the enhanced effectiveness of autografts is Concentrated Growth Factor (CGF), which comprises various proteins that stimulate osteogenesis and bone healing [18]. The incorporation of CGF into autografts significantly increases bone density and accelerates the regeneration process, as demonstrated by clinical outcomes, particularly in the treatment of mandibular fractures.

An experimental animal study by C. Black et al. supports these findings, showing that BMP-2, in combination with appropriate biomaterials such as collagen sponges, enables effective delivery of growth factors and stimulation of osteogenesis [19]. Furthermore, BMP-7 contributes to maintaining the balance between bone formation and resorption, which is essential for normal remodelling processes. A comparative experimental study by Y. Jin et al. highlighted that autografts also contain proteins that facilitate osteoconductivity, and their effectiveness is highly dependent on the carriers used to deliver recombinant growth factors like BMP-2 [20]. The study demonstrated that demineralised bone matrix (DBM) serves as a more effective carrier for BMP-2 than hydroxyapatite, offering improved bone regeneration and reduced fat infiltration. Thus, the presence of BMP-2 and other growth factors, in combination with optimal carriers, is critical for enhancing the osteoinductive properties of autografts. The effectiveness of autografts stems from the complex interplay of growth factors—including BMP-2, BMP-7, CGFs, and other signalling proteins—that stimulate osteogenesis, alongside the appropriate selection of carriers that ensure their delivery and activate local cellular regeneration mechanisms.

The primary advantage of autogenous bone grafts lies in their excellent biocompatibility, low risk of immune rejection, and strong potential for integration into host tissue. According to a clinical study by U. Tasdemir et al., outcomes of sinus lift and alveolar ridge augmentation surgeries using autogenous bone grafts were evaluated as excellent [21]. The regenerative capacity of autografts surpassed that of synthetic and xenogeneic substitutes, which was partly attributed to their ability to preserve osteocyte viability and stimulate vascularisation. Further enhancement of bone healing outcomes was achieved by combining autografts with growth factors, such as recombinant human vascular endothelial growth factor (rhVEGF).

Despite the superior biological outcomes associated with autogenous bone grafts, their use is limited by donor site morbidity, extended operative time, and postoperative pain. In a pilot study by U. Taşdemir et al., complications related to autogenous bone grafting were assessed, with the most significant being infection, prolonged healing time, and limited bone availability [22]. The authors confirmed that 20–30% of patients experience persistent donor site pain post-healing, with some reporting chronic pain or mild infection at the graft site. The study also highlighted the constraint posed by the limited volume of autografts, which restricts their application in large bone defect reconstructions—necessitating the use of allogeneic or synthetic substitutes in major surgical procedures.

Autograft harvesting is a widely practised technique in orthopedics and reconstructive surgery for the replacement of bone or soft tissue defects. However, this process carries the risk of donor site morbidity, which may manifest as pain, restricted mobility, infectious complications, or other functional impairments. Reducing these complications is essential to improving clinical outcomes and the postoperative quality of life. Scientific efforts are focused on optimizing harvesting techniques, selecting donor sites with lower risk, and identifying alternative sources of autologous tissue to minimize damage and accelerate rehabilitation. Accordingly, as noted in the systematic review and network meta-analysis by K. Kunze et al., minimizing donor site morbidity during autologous graft harvesting depends significantly on the type of graft selected [23]. In anterior cruciate ligament (ACL) reconstruction, the use of hamstring or quadriceps tendon autografts is associated with significantly lower donor site morbidity compared to the traditional bone–patellar tendon–bone graft. The choice of autograft should be individualized based on the patient’s level of physical activity and expectations, which reduces the likelihood of postoperative complications such as pain, mobility restriction, and functional loss. Furthermore, a systematic review and meta-analysis by A. Attia et al. emphasized that the technical aspects of bone autograft harvesting—such as from the calcaneus or the proximal and distal tibia—are crucial in reducing complications [24]. Selecting a donor site from the lower limb, rather than the iliac crest, significantly reduces the risk of chronic pain, infection, and neurological complications, which is critical for improving the quality of life of patients following surgery.

As highlighted in the clinical study by M.N. Khalid et al., the use of alternative autograft sources, such as the peroneus longus tendon, also contributes to minimizing donor site morbidity [25]. These methods provide favourable functional outcomes, faster recovery of mobility, and significantly reduced pain symptoms and risk of instability at the harvest site, making them promising candidates for widespread clinical application. Therefore, reducing donor site morbidity is achieved through a comprehensive approach that encompasses the selection of the optimal autograft type, appropriate donor site selection, refinement of surgical techniques, and the utilization of alternative tissues to decrease complication rates and enhance functional outcomes.

Autogenous bone grafts are widely applied in orthopedic, craniofacial, and maxillofacial surgery, where relatively rapid osseointegration and structural support are desired. A review article by H. Sun et al. described the use of guided bone regeneration (GBR) techniques in maxillofacial surgery, reporting predictable preservation of bone volume and excellent implant success rates [26]. In a study investigating autogenous dentin matrix grafts, the authors noted that in sites affected by severe periodontitis and similar conditions, these grafts performed as well as or better than xenogeneic materials. H. Sun et al. further observed that autogenous grafts combined with platelet-rich fibrin resulted in improved soft tissue healing and enhanced implant stability [26]. However, their findings also indicated that the success of autogenous grafts depends heavily on precise surgical handling and the patient’s bone metabolism, which may be influenced by factors such as age, systemic health, and lifestyle.

Thus, autogenous bone grafts remain the most effective material for bone regeneration, due to their inherent biocompatibility, the presence of viable cells, and the availability of growth factors that stimulate osteogenesis and remodelling. Nonetheless, their application is constrained by the limited availability of donor tissue and the risk of complications at the harvest site. This underscores the importance of optimizing harvesting techniques and exploring alternative graft sources. The appropriate selection of graft type, along with accurate surgical technique, can minimize complications and facilitate the successful restoration of bone structure.

#### 3.2.2. Allogeneic Bone Substitutes

The need for a large volume of bone is one of the scenarios in which allogeneic bone substitutes—obtained from human donors—serve as a viable alternative to autografts. These grafts are derived from donor tissues processed by bone banks through a screening and preparation process that removes cellular components while preserving elements of the tissue structure. Allogeneic and xenogeneic substitutes have demonstrated structural and osteoconductive advantages; however, they also carry risks of immune response and uncontrolled resorption, necessitating intensive processing methods to optimize their biological behaviour. In an in vitro experimental study, J. Liu et al. conducted laboratory tests to evaluate the effects of various configurations of allogeneic bone substitutes on cell adhesion and proliferation [27]. The researchers confirmed that combinations of bone pellets with bone powder exhibited superior osteoconductivity compared to cortical bone, due to the increased surface area and open architecture of these materials. The study also identified donor age and processing conditions as influential factors affecting graft quality, with bone from younger donors showing better mechanical properties and greater cellular compatibility. However, the findings demonstrated that although allogeneic grafts provide essential structural support and osteoconductivity, their lack of inherent osteogenic potential necessitates the addition of exogenous growth factors or viable cells to achieve optimal regeneration.

Current standards and protocols for donor screening and the processing of allogeneic bone tissue in bone banks are governed by national and international regulations that ensure graft safety, efficacy, and quality. Key regulatory frameworks include European Directives 2004/23/EC and 2006/86/EC, which outline requirements for donor selection, labelling, processing, storage, and transport of tissues—thereby minimizing the risk of infectious transmission and other complications [28,29]. As detailed in the experience report by I. Ilyas et al., these directives mandate comprehensive donor screening, which includes an in-depth medical history, serological testing (for HIV, hepatitis B and C, syphilis), and molecular diagnostics such as nucleic acid amplification tests (NAAT), all of which contribute to a thorough risk assessment [30].

Tissue processing protocols in bone banks involve multiple sequential steps: aseptic tissue procurement, sterile removal of soft tissues, disinfection, preservation (typically via cryopreservation or lyophilisation), and multi-level quality control measures, including microbiological and histological analyses. These procedures are comprehensively described in the review by A. Regmi et al. [31]. A critical component of these protocols is the implementation of a graft tracking system, which ensures full traceability of tissue movement from donor to recipient—an essential measure for transparency and for enabling a prompt response in the event of adverse outcomes.

The feasibility of implementing such stringent standards is supported by evidence demonstrating their effectiveness in significantly reducing the risk of infection transmission and other complications for recipients. These measures also enhance confidence in the use of allogeneic grafts and contribute to greater availability of high-quality materials for surgical interventions, as shown in a survey by T. Asamoto et al. [32]. For instance, the application of NAAT testing in compliance with modern requirements shortens the donor waiting period and decreases the number of rejected grafts caused by the serological window period, thereby increasing the efficiency of bone banks. Likewise, standardized sterilization and storage protocols reduce the risk of microbial contamination, which is particularly crucial for bone grafts due to their porous structure.

The implementation of international standards—such as the recommendations of the American Association of Tissue Banks (AATB)—alongside internal audit and quality control systems, adds another layer of safety and sustains a high level of trust among healthcare professionals and patients [33]. Collectively, these regulations and practices underpin modern bone grafting procedures, aiming to optimize clinical outcomes while minimizing associated risks.

Allogeneic bone undergoes extensive processing, including sterilization, decalcification, and demineralisation, to mitigate the risks of disease transmission and immune rejection. A review by I. Um et al. examined the osteoinductivity and antigenicity of allogeneic demineralised dentin matrix (Allo-DDM), a dentin-derived allograft [34]. The study found that decalcification methods enhanced the release of bone morphogenetic proteins (BMPs), thereby increasing osteoinductivity. Low antigenicity values were achieved through rigorous sterilization protocols that preserved mechanical integrity. The preservation of collagen structures and growth factors through high hydrostatic pressure (HHP) sterilization was shown to be superior to conventional techniques such as gamma irradiation or thermal treatment, which may compromise the structural strength of the bone matrix. However, the authors noted that, despite these optimized processing methods, allografts remain less bioactive than autografts. Consequently, biological augmentation strategies—such as the incorporation of growth factors or the seeding of stem cells—remain important avenues for improving the regenerative potential of allogeneic grafts.

Allogeneic bone substitutes are widely used in fracture healing, spinal fusion, and dental bone grafting—particularly in cases where large volumes of graft material are required. A preclinical study by P. Tournier et al. [13] demonstrated that deproteinised allografts serve as effective scaffolds for stem cells and promote bone formation through natural tissue regeneration mechanisms. The preservation of collagen and mineral structure enhances cell adhesion and proliferation, which is especially valuable in revision surgeries and the reconstruction of extensive bone defects.

However, allogeneic grafts also present notable limitations. An experimental study on mechanical properties conducted by J. Waletzko-Hellwig et al. found that although high-pressure-treated allografts retained structural integrity, they exhibited only limited capacity to induce natural bone regeneration when compared to autografts [35]. The absence of viable osteogenic cells renders these grafts dependent on a slow remodelling process mediated by the host, which may result in the formation of fibrous tissue rather than functional bone. These limitations underscore the need for innovative tissue engineering strategies, such as combining allografts with stem cells and bioactive surface coatings.

Allogeneic bone substitutes derived from human donors therefore offer crucial structural support and osteoconductive properties in the reconstruction of large bone defects. Modern processing protocols—including sterilization and demineralisation—help to minimize the risk of infection transmission and adverse immune responses. Nevertheless, due to their limited osteogenic potential, there is growing interest in enhancing these materials through the incorporation of growth factors and stem cells, with the aim of improving their regenerative capacity and clinical outcomes.

#### 3.2.3. Xenogeneic Bone Substitutes

Xenogeneic bone substitutes—derived from bovine, porcine, and equine sources—are widely used in orthopedics and maxillofacial surgery for various procedures, including alveolar ridge augmentation and sinus lift. An experimental animal study by A. Shao et al. investigated the immunological response to xenogeneic bone matrices [36]. Additionally, a systematic review of in vitro studies by R. Amid et al. found that bovine xenografts possess a mineral structure closely resembling that of human bone, making them effective scaffolds for osteoconduction [37]. Porcine variants, meanwhile, demonstrated superior remodelling properties due to their enhanced resorption capacity. Despite these advantages, xenogeneic materials require thorough processing to remove cellular components and minimize immunogenicity. Furthermore, their prolonged resorption period can delay natural healing, often necessitating the adjunctive use of growth factors or bioactive surface coatings to enhance integration with recipient tissues. To systematize the comparative characteristics of xenogeneic materials of differing origins, a detailed analysis of their structural features, clinical advantages, and limitations is presented in Table 2.

The presented data demonstrate that the selection of a xenogeneic material source is determined by the balance between structural stability and the speed of biological integration. Bovine-derived materials provide optimal long-term support for reconstructive procedures in dentistry, where volumetric stability is essential. Porcine substitutes offer advantages in clinical situations requiring accelerated remodelling, particularly in periodontology and pediatric applications. Equine materials occupy a niche position as alternatives in cases of cultural or religious restrictions, or in patients with individual intolerance. The key factor in material selection remains not only biological effectiveness, but also the ethical and cultural acceptability for the individual patient.

Xenotransplantation—the transplantation of cells, tissues, or organs between species—carries the risk of transmitting infectious diseases, particularly zoonoses, which may pose serious threats to both recipient health and public safety. Pathogens potentially transmissible through xenografts include viruses such as porcine cytomegalovirus (PCMV/PRV), retroviruses including porcine endogenous retroviruses (PERVs), and various species-specific agents such as hepatitis viruses, as outlined in the expert review by H. Groenendaal et al. [40,41]. The risk of viral transmission from animals to humans, with the possibility of novel infectious disease emergence, requires rigorous oversight and comprehensive risk management. Current protocols for minimizing the risk of zoonotic transmission are highly effective due to a multifaceted approach. This includes mandatory testing of donor animals using polymerase chain reaction (PCR) and nucleic acid amplification tests (NAAT) for a comprehensive panel of known pathogens, following recommendations from regulatory bodies such as the U.S. Food and Drug Administration and the European Medicines Agency [42,43]. Additionally, multi-stage processing of graft materials using combined physical and chemical sterilization techniques, as outlined by Hawthorne et al., is standard practice [44]. Long-term post-operative monitoring of recipients further enhances safety, also in accordance with the protocols established by Hawthorne and colleagues. The effectiveness of these safety measures is evidenced by the absence of documented cases of PERV or other serious zoonotic disease transmission in clinical practice involving properly processed xenogeneic bone substitutes. This supports the adequacy of current safety standards, provided that all processing and monitoring protocols are strictly followed.

Biocompatibility and minimisation of immune response are critical for the clinical success of xenografts. A systematic review by L. Sánchez-Labrador et al. confirmed that hydrothermal and chemical processing techniques effectively remove immunogenic proteins while preserving the structural integrity of the mineralised matrix [45]. This preservation is crucial for ensuring the biocompatibility and osteoconductivity of the material. Furthermore, the review highlighted that the incorporation of osteoinductive agents—such as bone morphogenetic proteins (BMPs) or mesenchymal stem cells—significantly enhances the functionality and integration of xenografts in clinical applications.

An experimental animal study by A.T. Jerbić Radetić et al. highlighted the widespread use of xenografts in orthopedic surgery, dental implantology, and periodontal regeneration, particularly in cases where the application of autografts or allografts is restricted by volume limitations or patient preferences [38]. A preclinical in vivo study by A. Pröhl et al. demonstrated that xenografts suspended in hyaluronic acid achieved excellent integration and bone ingrowth, albeit with slower remodelling compared to autologous materials [39]. The researchers also observed that combining xenografts with bioactive components—such as platelet-rich fibrin or stem cell-based scaffolds—promotes enhanced vascularisation and accelerates healing processes.

Thus, xenogeneic bone substitutes derived from bovine, porcine, and equine tissues are widely applied in orthopedic, maxillofacial, and dental surgical procedures. Bovine-derived materials offer an optimal osteoconductive scaffold due to their structural similarity to human bone, while porcine-derived materials exhibit superior remodelling characteristics. These grafts require complex processing techniques—including hydrothermal, chemical, and genetic engineering methods—to eliminate immunogenic components and reduce the risk of zoonotic disease transmission. The regenerative efficacy of xenogeneic substitutes is significantly enhanced when combined with osteoinductive agents, such as bone morphogenetic proteins or mesenchymal stem cells, which improve tissue integration and vascularisation, compensating for their relatively slow remodelling. Systematic evaluation of clinical applications suggests that xenografts are ideally suited for dental implantology and small bone defects, where their slow resorption offers long-term structural stability. However, they are not recommended for critical load-bearing applications or for large defect reconstruction in younger patients, due to their limited osteogenic potential. The most favourable outcomes are achieved when xenografts are used in conjunction with autogenous bone or growth factors, enabling the combined advantages of both material types to be realized.

#### 3.2.4. Synthetic Bone Substitutes

Synthetic bone grafts play a critical role in regenerative medicine by providing structural support at the defect site while simultaneously serving as a biologically favourable substrate that facilitates osteoblastic adhesion, proliferation, and subsequent induction of new bone formation. Beyond their mechanical and osteoconductive properties, these grafts can be further functionalized as delivery system for bioactive molecules, including growth factors and therapeutic drugs, thereby enhancing their regenerative potential and broadening their clinical applications. Among synthetic bone grafts, the most extensively studied biomaterials include calcium phosphate–based cements, calcium phosphate ceramics, calcium sulphate, bioactive glasses, and polymers [1].

Calcium phosphate cements (CPCs) present several advantages: they are bioactive, suitable for large-scale manufacturing, easy to handle, and injectable, which allows them to adapt to irregularly shaped bone defects. Unlike autogenous and allogeneic grafts, CPCs do not carry risks such as donor site morbidity or infection. Moreover, their biocompatibility and compositional similarity to bone make them highly suitable candidates for bone regeneration.

Synthetic bone substitutes based on calcium phosphate ceramics—particularly hydroxyapatite (HA) and β-tricalcium phosphate (β-TCP)—are characterized by controlled degradation rate, and structural similarity to natural bone. HA is associated with excellent biocompatibility, as it does not provoke local or systemic toxicity, inflammatory responses, or adverse immunological reactions, thereby reinforcing its value as a clinically reliable bone substitute material. Beta-tricalcium phosphate (β-TCP), typically sintered at temperatures below approximately 1125 °C, offers the advantage of thermodynamic stability within the biological environment, while demonstrating greater resorbability than hydroxyapatite under physiological conditions.

In an experimental study by E. Maevskaia et al., the osteoconductive properties of 3D-printed ceramic scaffolds were examined, and it was found that the efficiency of bone tissue adhesion and cell proliferation is directly influenced by the surface microarchitecture and optimal porosity of the material [46]. Structures featuring triply periodic minimal surfaces (TPMS) proved especially promising, offering enhanced mechanical strength, vascularisation, and structural stability. Despite these advantages, synthetic materials possess limited osteoinductive potential, which necessitates their combination with bioactive growth factors or stem cells to achieve optimal integration with host tissues.

3D printing of ceramic frameworks presents significant advantages over traditional manufacturing methods due to its ability to produce complex, patient-specific structures with precisely controlled porosity—thereby promoting improved vascularisation and tissue ingrowth. As noted in the review by Y. Ding et al., this technology also reduces production time and enhances implant precision [47]. However, the technology review by X. Li et al. highlighted that 3D printing of ceramics remains costly and technically demanding, with current limitations in mechanical strength and production scalability [48]. Consequently, although 3D printing represents a highly promising direction, it still requires further development before it can be widely adopted in clinical settings.

Calcium sulphate, one of the earliest synthetic bone graft substitutes, has long been employed in orthopedic and dental applications due to its biocompatibility and ability to completely resorb in vivo. Its rapid degradation profile allows for temporary defect filling and space maintenance, often serving as a carrier for antibiotics or growth factors. However, its primary limitation lies in the imbalance between its fast resorption rate and the relatively slower pace of new bone formation, which can result in incomplete defect regeneration if not combined with other biomaterials. It has been extensively incorporated into composite formulations together with hydroxyapatite, tricalcium phosphate, calcium carbonate, and monocalcium phosphate monohydrate. These mixtures are often designed as injectable pastes that can harden in situ within bone defects, offering a minimally invasive approach to defect stabilization and filling [4].

Biodegradable polymers—such as polylactic acid (PLA), polycaprolactone (PCL), and collagen composites—constitute another promising class of synthetic bone substitutes, owing to their moulding properties and adaptive degradation characteristics. These polymers are readily biodegradable in vivo; however, their limited mechanical strength and inherently low osteoconductivity restrict their application as standalone scaffolds for bone regeneration [1]. In an experimental study by I. Nifant’ev et al., polyester–mineral composites were investigated, revealing that the inclusion of β-tricalcium phosphate or carbonated hydroxyapatite significantly enhanced the mechanical strength and biocompatibility of the materials [49]. The researchers concluded that these composites offer controlled degradation, which is particularly valuable in anatomical areas where gradual bone remodelling is required.

Bioactive glass constitutes a distinct and important class of synthetic bone substitutes, characterized by its ability to establish direct bonds with host bone tissue while releasing biologically active ions that stimulate cellular responses. A critical feature of BGs is their surface reactivity, which closely mimics the chemical and structural properties of the bone mineralization phase, thereby supporting bone reconstruction. Notably, the bioactivity and resorption kinetics of BGs are strongly dependent on their chemical composition, which in turn governs their biological performance and clinical applicability [1]. In an experimental study investigating mechanical properties, T.Y. Kang et al. demonstrated that modification of bioactive glass with zirconium oxide (ZrO_2_) significantly enhances its mechanical strength while preserving its bioactivity [50]. The modified materials exhibited improved cellular responses, elevated alkaline phosphatase activity, and accelerated mineralization—making them particularly suitable for applications in bone tissue engineering.

A comparative analysis of the three principal groups of synthetic bone substitutes reveals distinct advantages and limitations relevant to different clinical contexts. Calcium phosphate ceramics—such as hydroxyapatite and β-tricalcium phosphate—exhibit the highest osteoconductivity among synthetic materials due to their chemical resemblance to the mineral phase of natural bone. This makes them particularly well-suited for filling small defects and for augmentation procedures in dentistry. Biodegradable polymers, including polylactic acid (PLA) and polycaprolactone (PCL), offer the benefit of controlled degradation in parallel with new bone formation, which is particularly valuable in pediatric applications and in cases where gradual material replacement with native tissue is desired. Bioactive glass, on the other hand, is notable for its active stimulation of bone formation via the release of silicon and calcium ions. Among synthetic materials, it demonstrates the highest rates of vascularisation, making it especially promising for complex cases involving compromised blood supply. However, a common limitation shared by all three synthetic groups is their insufficient osteoinductive potential. As a result, these materials often require biological augmentation—such as the addition of growth factors or stem cells—to achieve effective integration and regeneration, in contrast to autogenous grafts, which possess inherent biological activity.

In summary, some of the synthetic bone substitutes include calcium phosphate ceramics, biodegradable polymers, and bioactive glass. These materials offer controlled degradation, excellent biocompatibility, and the potential for custom implant fabrication via 3D printing. Despite their favourable osteoconductive properties, their limited osteoinductive capacity necessitates combination with bioactive components for optimal performance in clinical settings. To consolidate and compare the key properties of all bone substitute categories discussed, a systematic overview is provided in Table 3.

The analysis of the comparative characteristics of bone substitutes reveals a fundamental contradiction between biological effectiveness and the practical availability of materials in modern regenerative medicine. The presented data indicate an inverse relationship between osteogenic potential and the clinical accessibility of different types of substitutes. Autogenous grafts demonstrate the highest values for all three key biological parameters—osteogenicity, osteoinductivity, and osteoconductivity—owing to the preservation of viable osteoblasts and endogenous morphogenetic proteins. However, their clinical use is constrained by the limited volume of available donor tissue and a statistically significant incidence of donor site complications, which, according to the analyzed studies, affects 20–30% of patients.

Allogeneic and xenogeneic substitutes represent the opposite end of this spectrum. While they offer virtually unlimited availability due to standardized donor sourcing and processing, they entirely lack osteogenic properties as a result of decellularisation and sterilization procedures. Their moderate osteoconductivity is retained through the preservation of the mineralised matrix; however, the absence of viable cells renders their efficacy heavily dependent on the host’s endogenous regenerative capacity. Synthetic materials occupy a distinct niche, offering high biocompatibility and enabling precise control over physicochemical parameters such as porosity, degradation rate, and mechanical strength. Nevertheless, their biological inertness necessitates functionalisation with osteoinductive molecules or the incorporation of cellular components to achieve satisfactory regenerative outcomes.

This identified heterogeneity in functional characteristics underscores the necessity for a differentiated approach to bone substitute selection. Such an approach must be based on multifactorial analysis, taking into account anatomical and topographic features of the defect, biomechanical loading conditions, the patient’s metabolic status, and their potential for endogenous regeneration. A promising direction to address existing limitations is the development of next-generation composite biomaterials. These aim to integrate the structural and mechanical advantages of synthetic scaffolds with the biological activity of natural growth factors and cellular elements. Such a synergistic strategy holds the potential to enhance osteogenesis significantly, thereby improving clinical outcomes in regenerative bone medicine.

### 3.3. Biological and Mechanical Properties of Bone Substitutes

The porosity of a bone substitute is a determining factor in bone integration and vascularisation and plays a significant role in overall functionality. Porous structures promote osteoconduction by providing a conducive environment for the infiltration, attachment, and proliferation of bone cells within the scaffold, ultimately facilitating bone regeneration and remodelling. L. Cheng et al. investigated the impact of micro- and nanoscale porosity on the performance of bone substitutes and highlighted the necessity of highly porous structures to support nutrient diffusion and osteoblast adhesion [52].

Modern manufacturing methods for bone substitutes aim to achieve maximum precision in creating porous architectures, as pore size and interconnectivity directly influence osteointegration, vascularisation, and the remodelling process. One key approach involves solid-state sintering technologies in combination with porogenic agents, such as ammonium bicarbonate. This method enables the formation of a controlled porous structure with pore sizes ranging from 23 to 210 μm. As emphasized by D. Liang et al., the pore size and shape can be finely tuned by selecting specific porogen granule sizes, achieving a high degree of interconnectivity while maintaining mechanical integrity [53].

Another highly accurate technique for controlling pore geometry involves the use of micro-computed tomography (micro-CT) combined with digital image processing algorithms. In particular, the fuzzy distance transform method allows for detailed assessment not only of pore diameter, but also shape, surface area ratio, and permeability—parameters critical for modelling nutrient diffusion and cellular infiltration. According to M.P. Ferraz et al. [51], this approach facilitates the design of porous structures with precise characteristics without compromising biomechanical performance, thereby enabling more effective, patient-specific implant design. Frameworks produced via 3D printing using triply periodic minimal surfaces and hierarchical pore organization demonstrate significantly enhanced osteoconductivity compared to traditional dense materials. However, excessive porosity leads to reduced mechanical strength, limiting their suitability for load-bearing applications. To address this limitation, L. Cheng et al. [52] proposed composite strategies whereby biodegradable synthetic polymers are used to reinforce porous ceramics, achieving an optimal balance between mechanical strength, porosity, and bioactivity.

To further improve this balance, functionally graded materials combining polymers—such as polycaprolactone (PCL) or poly (lactic-co-glycolic acid) (PLGA)—with β-tricalcium phosphate (β-TCP) have been employed, as demonstrated in the study by A. Kumar et al. [54]. In such systems, porosity gradually increases through polymer degradation while mechanical strength is maintained through concurrent new bone formation. Another approach, proposed by D. Celik and C.B. Ustundag, involves the hybrid design of ceramic frameworks featuring both cortical and trabecular architectures [55]. This configuration provides approximately 70% open porosity while maintaining mechanical strength comparable to natural bone. A comparative analysis of the biomechanical properties of major synthetic bone substitute types reveals a wide spectrum of performance characteristics, supporting the selection of optimal materials for specific clinical applications. These data are summarized in Table 4.

The presented data confirm an inverse relationship between porosity and the mechanical strength of bone substitute materials. The highest compressive strength is demonstrated by bioactive glass modified with zirconium oxide (up to 140 MPa) at moderate porosity levels (40–60%), whereas highly porous polymer composites (70–85%) show significantly lower strength values (5–25 MPa). Degradation rates also vary widely: from 4 to 8 weeks for calcium sulphate to more than 24 months for hydroxyapatite, defining their clinical application range from short-term drug delivery to long-term structural support, respectively. β-Tricalcium phosphate and hybrid ceramic frameworks demonstrate an optimal balance of properties, combining moderate compressive strength (15–40 MPa) with controlled degradation (6–18 months), making them versatile materials suitable for a broad spectrum of clinical scenarios.

A central challenge in the development of synthetic bone substitutes is achieving an appropriate balance between degradation rate and mechanical properties. Scaffolds must provide sufficient initial structural support while allowing for a gradual transition to regenerated bone. G. Marongiu et al. tested synthetic materials based on calcium sulphate and β-tricalcium phosphate in an osteoporotic bone defect model [56]. Their findings showed that β-TCP exhibited a degradation profile well aligned with new bone formation, supporting long-term structural integrity. In contrast, calcium sulphate resorbs too rapidly to be effective for load-bearing or long-duration applications, making it suitable only for short-term use. The in vivo degradation rate of bone substitutes is influenced by multiple factors, including pH, enzymatic activity, material porosity, and cellular responses. As demonstrated by H. Kang et al., multichannel structures enhance permeability and promote faster degradation through increased ion exchange [57]. Similarly, K. Kowalewicz et al. found that the level of osteoclast activity in the implantation zone significantly accelerates the resorption of the graft material [58].

Biocompatibility remains a critical requirement for synthetic bone substitutes, as clinical success depends on minimizing immune responses and preventing fibrous encapsulation. M. Heitzer et al. evaluated the biocompatibility of alloplastic, allogeneic, and xenogeneic bone substitutes combined with human dental pulp stem cells (DPSCs) and extracellular vesicles (EVs) [2]. The study showed that EVs derived from stem cells exhibited the greatest potential for promoting osteogenic differentiation, supporting the use of biological enhancements to improve graft integration. Although biphasic calcium phosphate (BCP) is characterized by good biocompatibility, M. Heitzer et al. noted that it lacks intrinsic osteoinductive capacity [59]. Therefore, it requires the addition of growth factors or seeded cells to effectively initiate bone regeneration. The authors concluded that more effective bone healing outcomes are achievable with next-generation bone substitutes that combine biocompatible scaffolds with bioactive molecules, providing a synergistic effect in stimulating osteogenesis.

Therefore, porosity is a critical factor in determining the effectiveness of synthetic bone substitutes, as it directly influences osteoconduction, vascularisation, and cellular infiltration. Modern manufacturing technologies—such as 3D printing and the incorporation of porogenic agents—enable precise control over pore size and interconnectivity, thereby optimizing biological performance. A persistent challenge remains the need to balance porosity with mechanical strength, which is being addressed through the development of functionally graded materials and composite scaffold structures. The degradation rate of the material should be synchronized with the pace of natural bone regeneration, with β-tricalcium phosphate exhibiting particularly favourable characteristics for long-term applications. To enhance biocompatibility and osteoinductive potential, synthetic substitutes are increasingly combined with bioactive molecules, stem cells, or extracellular vesicles—an approach that significantly improves their clinical effectiveness and regenerative outcomes.

### 3.4. Clinical Applications in Regenerative Medicine

In orthopedic traumatology, bone substitutes are employed for fracture healing, defect repair, and joint reconstruction. I. Sallent et al. demonstrated the clinical efficacy of bioactive scaffolds, ceramics, and engineered allografts, emphasizing the particular utility of synthetic substitutes based on calcium phosphate ceramics and bioactive glass in the management of load-bearing bone defects [60]. The researchers also highlighted the potential of tissue-engineered grafts incorporating mesenchymal stem cells and growth factors, which enhance vascularisation and promote superior regenerative outcomes compared to traditional grafting methods.

In revision surgery, bone substitutes are used to restore bone stock and improve implant integration. L. Massari et al. evaluated the clinical safety of a porous hydroxyapatite substitute in a cohort of 114 patients, confirming excellent biocompatibility and mechanical stability without notable side effects [61]. However, the authors underscored the inherent lack of osteogenic potential in hydroxyapatite-based materials, which necessitates the incorporation of bioactive agents to optimize regenerative performance.

In maxillofacial surgery and dental practice, bone substitutes are commonly applied in alveolar ridge augmentation, sinus lift procedures, and implant site preparation. B. Mahardawi et al. reported that allogeneic demineralised dentin matrix (Allo-DDM) was associated with low postoperative complication rates and sustained long-term functionality, without the morbidity associated with autologous donor sites [62]. C. Batra et al. investigated the combination of recombinant human platelet-derived growth factor-BB (rhPDGF-BB) with xenogeneic substitutes and demonstrated improved soft tissue regeneration and enhanced preservation of bone volume [63].

In neurosurgery, bone substitutes are applied in procedures such as cranioplasty, spinal fusion, and the reconstruction of defects following tumour resection. S. Zhang et al. investigated the use of autogenous dentin material for the reconstruction of cranial defects, confirming its high osteoconductive and osteoinductive potential due to its compositional similarity to natural bone matrix [64]. K.J. Jung et al. demonstrated the clinical success of multi-channel granular calcium phosphate substitutes in spinal fusion procedures, reporting long-term mechanical stability and high union rates [65]. A systematization of clinical experience regarding the use of bone substitutes across different medical specialties is presented in Table 5, which outlines the specific clinical requirements and observed outcomes within each field.

### 3.5. Three-Dimensional Bioprinting and Future Directions in Bone Substitution

Modern advances in 3D bioprinting enable the fabrication of individualized tissue substitutes with precise control over porosity and the incorporation of bioactive coatings. The scaffold, a three-dimensional and highly porous substrate, is a key element in tissue engineering. After expansion in culture, cells are transferred onto the scaffold, where they adhere, proliferate, remain viable, and generate the extracellular matrix composed of structural and functional proteins and saccharides that form living tissue. The material composition of the scaffold, together with its internal architecture (including the dimensions of struts, walls, pores, or channels), regulates and controls the biological behaviour of the cells [69]. Presentation of common bioprinting techniques is provided according to the description of Li et al. [70].

- Extrusion-based bioprinting

Extrusion-based bioprinting relies on the continuous deposition of bioinks via pneumatic, piston, or screw-driven systems. Techniques such as semi-solid extrusion (SSE) and fused deposition modelling (FDM) enable fabrication of both soft tissue analogues and bone-mimetic scaffolds. Natural polymers (alginate, gelatin, collagen, nanocellulose) and synthetic polymers (polycaprolactone [PCL], polyvinyl alcohol [PVA]) can be blended with growth factors or nanoparticles to optimize printability and cellular responses. Advantages include the ability to print high-viscosity bioinks and create mechanically robust structures, whereas lower spatial resolution and shear-induced cell stress remain challenges [71].

Looking forward, future developments are expected to focus on the design of next-generation bioinks with tunable viscoelasticity, the integration of biologically active nanomaterials to guide cellular behaviour, and the advancement of multi-scale fabrication strategies that reconcile structural precision with mechanical robustness. Such innovations are anticipated to expand the applicability of extrusion-based bioprinting to complex, clinically relevant constructs for tissue engineering and personalized medicine.

- Inkjet-Based Bioprinting

Inkjet bioprinting is a non-contact, droplet-based technique that employs thermal, electrostatic, or piezoelectric actuation to deposit cell-laden bioinks with high spatial precision. Its primary advantage lies in rapid, cost-effective fabrication and compatibility with low-viscosity natural and synthetic polymers such as alginate, gelatin, collagen, chitosan, and polyethylene glycol (PEG). While it allows high-throughput printing, limitations include restricted bioink viscosity and lower cell density tolerance, which can affect the physiological relevance of printed constructs [72]. 

Collectively, inkjet-based strategies hold considerable promise for tissue engineering and regenerative medicine applications, although limitations associated with bioink viscosity and cell density continue to present challenges that warrant further optimization.

- Pressure-assisted bioprinting

Pressure-assisted bioprinting extrudes bioinks under controlled pressure at room temperature, enabling the direct embedding of viable cells. Materials include hydrogels, collagen, chitosan, ceramics, and synthetic polymers such as PEG, PLA, and PCL. The method allows for high-viscosity material printing and customization of construct architecture, though shear stress, bioink optimization, and resolution limitations require careful consideration [70].

Future directions in PAB are likely to focus on engineered bioinks with tunable viscoelastic properties, multi-material printing for hybrid tissue constructs, and integration with post-processing strategies such as crosslinking or mineralization to enhance mechanical and functional properties. These advances aim to broaden the applicability of pressure-assisted bioprinting in tissue engineering, regenerative medicine, and personalized implant fabrication.

- Laser-assisted bioprinting

Laser-assisted bioprinting uses a pulsed laser to transfer bioinks onto a substrate in a droplet-based, non-contact manner. Bioinks comprise natural polymers (collagen, gelatin, alginate, fibrin), synthetic hydrogels (PEG), decellularized extracellular matrix (ECM), nano-hydroxyapatite, and cell-laden formulations. LAB provides high spatial resolution, precise cell placement, and non-contact deposition, but faces challenges related to equipment cost, laser-induced thermal stress, and bioink viscosity constraints [70].

Future directions for LAB involve the development of next-generation bioinks with enhanced optical and rheological properties, integration with multi-material printing strategies, and the exploration of tissue-specific composite formulations for regenerative medicine and personalized tissue engineering applications. Table 6 presents comparative overview of 3D bioprinting modalities.

L. Cheng et al. demonstrated that scaffolds composed of hydroxyapatite, β-tricalcium phosphate (β-TCP), and bioactive polymers exhibit enhanced osteoconductivity and excellent resistance to mechanical loads [52]. Three-Dimensional bioprinting technology offers unprecedented control over scaffold architecture: porosity can be modulated within the range of 30–90%, with pore size precision of ±50 μm. Printing resolution ranges from 100 to 200 μm depending on the technology used (stereolithography, selective laser sintering, or extrusion printing). Additionally, it allows for the creation of complex internal geometries, including gradient structures and interconnected channel systems with diameters of 150–500 μm to facilitate vascularisation. Compared with traditional implantation methods, 3D bioprinting offers critical advantages: personalisation based on patient-specific anatomy derived from computed tomography data, integration of multiple materials within a single construct, controlled spatial distribution of bioactive molecules, and the fabrication of functionally graded scaffolds that mimic the transition from cortical to cancellous bone. However, the technology presents significant limitations, including high equipment costs (ranging from $100,000 to over $1 million for industrial systems), lengthy fabrication times for complex structures (6–24 h depending on size and complexity), a limited range of clinically certified biocompatible materials, and the requirement for post-processing (e.g., sintering, sterilization), which may affect mechanical integrity and bioactivity. The integration of stem cells with bioprinted scaffolds represents a major advancement in regenerative medicine. An experimental study by Meesuk L. et al. reported that both 3D-printed HA and calcium phosphate-coated 3D-printed HA supported the proliferation and osteogenic differentiation of BM-MSCs and UC-MSCs [67]. A clinical study by P. Adamska et al. confirmed the therapeutic efficacy of off-the-shelf stem cell therapies in geriatric patients, offering regeneration without the need for autologous bone harvesting [68].

In 2023 Hao and colleagues reported a clinical case of bone defect repair using a self-developed 3D bioprinted active bone [73]. To restore the left tibia, they fabricated a bone scaffold from a polycaprolactone/β-tricalcium phosphate (PCL/β-TCP) composite integrated with bio-ink derived from the patient’s own platelet-rich plasma (PRP).

Scaling and commercializing bioprinted tissues face major challenges. Designing, sourcing cells, and manufacturing are often highly personalized, which drives up costs. In the short term, companies may focus on universal scaffolds or components that reduce the need for individualized materials. Acellular scaffolds seeded with a patient’s own cells could lower costs while limiting rejection risks. Although personalized 3D bioprinting requires high upfront investment, it may prove more cost-effective than long-term non-curative treatments such as dialysis or diabetes management. In many cases, a single intervention is economically preferable to lifelong therapies.

One of the central obstacles in bioprinting is achieving an optimal balance between scaffold degradability and mechanical functionality. Controlling the degradation rate is crucial, since overly rapid degradation can compromise structural support, whereas excessively slow degradation may induce inflammatory responses, thereby hindering tissue regeneration [74]. In parallel, ensuring appropriate mechanical performance requires careful evaluation of multiple parameters—including compressive and tensile strength, elasticity, and fatigue resistance—throughout scaffold fabrication [75].

Hydrogels, which represent water-swollen porous networks, have emerged as promising candidates for cell encapsulation, tissue engineering, and 3D bioprinting applications. For successful use in bioprinting, hydrogels are required to exhibit tunable substrate stiffness and the ability to undergo network reconfiguration post-printing, thereby enabling critical cellular processes such as spreading, migration, proliferation, and intercellular interaction. Despite these advantages, their inherently poor mechanical properties remain a major limitation [75].

Stem cells are considered the preferred seed cells in 3D bioprinting owing to their capacity for self-renewal and multilineage differentiation, as well as their genetic compatibility, which reduces the risk of immune rejection. Nevertheless, their declining proliferative ability upon differentiation limits their use in large 3D constructs. Induced pluripotent stem cells (iPSCs) offer an alternative source, but their generation is complex, time-consuming, and associated with risks such as epigenetic abnormalities and tumorigenicity. Another approach is the use of differentiated cell types for tissue and organ modelling; however, this strategy is costly, technically demanding, and hampered by low differentiation efficiency [76].

3D-bioprinted tissues and organs face major regulatory challenges. While the FDA currently applies the same rules to printed and conventional devices, the 21st Century Cures Act allows some regenerative medicine therapies to qualify for RMAT status, including cell and tissue therapies, engineered constructs, and gene-based interventions. Regulating functional bioprinted products is difficult because of their complex mechanisms, multiple active components, and uncertain long-term effects. Even small manufacturing changes may alter product properties unpredictably, underscoring the need for clear and centralized regulatory pathways, especially as hospitals and research institutions produce personalized devices. Current FDA guidelines and international efforts acknowledge these issues, while standardization and technological convergence could help address them. To speed clinical translation, thorough safety and efficacy testing must be combined with clear instructions for use, defining purpose, patient population, and conditions of application. This structured approach could simplify approval and enable faster patient access to bioprinted therapies, improving healthcare outcomes [77].

The analysis of clinical applications reflects an evolution from simple structural substitutes towards sophisticated bioactive systems with osteoinductive capabilities. Each medical specialty presents distinct requirements for bone substitutes, necessitating the design of specialized materials and technologies. The most promising outcomes are observed when synthetic scaffolds are integrated with biological components, achieving an optimal balance between structural support and regenerative functionality. Nonetheless, clinical practice reveals a spectrum of potential complications. With autografts, donor site morbidity ranges from 20 to 30%, including persistent pain and infection risk [22]. Allogeneic and xenogeneic materials, despite advanced processing, still pose risks of infection transmission and immune reaction. Synthetic substitutes may result in fibrous capsule formation due to their lack of intrinsic osteoinductivity. The use of BMP-2 has been associated with ectopic bone formation and local inflammation in 15–20% of cases. Furthermore, 3D-printed implants present risks of mechanical failure under excessive loading and susceptibility to bacterial colonization of porous structures.

In recent years, bone regeneration has benefited from the development of novel biomaterials (such as energetic materials, metabolic regulatory materials, hydrogels, etc.) that go beyond traditional autogenous, allogenic, and synthetic options. A growing body of evidence highlights the crucial role of bioelectric signals and cellular bioenergetics (CBE) in tissue repair.

Electrical stimulation can enhance the healing of nerves, bones, cardiovascular tissues, and wounds, yet conventional metal electrode devices are invasive and rely on external power sources. Consequently, biocompatible electrically active materials have emerged as promising alternatives, offering the potential to modulate bioelectric cues in situ. At the same time, tissue regeneration is tightly linked to CBE, as enhanced metabolic states promote anabolic biosynthesis and mitosis, thereby accelerating repair. Integrating these bioelectric and bioenergetic strategies through advanced biomaterials may provide a synergistic approach for next-generation bone repair therapies. Haoming Liu et al. develop strategy to produce a bioenergetic scaffold that is simple and easy to implement for enhanced bone regeneration [78].

Recent studies suggest that the intrinsic properties of synthetic materials can influence cell metabolism and potentially guide cell behaviour to affect regenerative engineering outcomes. This can occur through the release of soluble metabolic regulatory factors (such as ions, degradation products, and oxygen), the incorporation of antioxidative properties, and the fine-tuning of cell adhesion, chemical composition, topography, and material stiffness [79]. Metabolic regulation of energy, oxygen, and redox homeostasis plays a key role in controlling cell behaviour during differentiation, angiogenesis, and immune responses in regenerative engineering. Cells can also sense and respond to extracellular cues—such as adhesivity, topography, and stiffness—through mechanosensing networks, influencing their metabolic and biosynthetic states. Although still an emerging field, leveraging metabolic regulation in biomaterials design holds promise for both cell therapy and advanced scaffold development. For instance, citrate-based biomaterials with tunable properties could dynamically provide or absorb metabolic cues to optimize regeneration. Integrating material-driven metabolic pathways offers a unique opportunity for interdisciplinary collaboration to create biomaterials that actively guide cell metabolism and behaviour for predictable regenerative outcomes [79].

With the advancement of medical technology, bioactive materials have become a central focus in promoting bone repair, with hydrogels emerging as one of the most prominent biomaterials in bone tissue engineering (BTE). Hydrogels offer high biocompatibility and mimic the structural characteristics of the porous extracellular matrix (ECM), providing an in vivo environment conducive to cell survival and facilitating bone regeneration. Injectable hydrogels are particularly advantageous, as they can conform to irregular or large defects, filling the cavity while maintaining granule cohesiveness during and after delivery. Various hydrogel matrices have been developed in recent years, enhancing osteogenic induction and improving scaffold efficacy in the treatment of complex bone defects arising from trauma, tumour resection, or infection [80,81].

Composite scaffolds, combining hydrogels with organic or inorganic fillers, further enhance mechanical and biological performance. Examples include poly(ethylene glycol) diacrylate with clay, Oligo(poly(ethylene glycol) fumarate) with calcium phosphate, cyclic acetal hydrogels with nano-hydroxyapatite, alginate with 45S5 bioactive glass, gelatin methacrylate with hydroxyapatite, elastin-like polypeptide collagen with 45S5 bioactive glass, and carbon nanotube-based systems. The primary goal of these composites is to maintain structural integrity during injection and implantation, while the in situ gelation ability of hydrogels—triggered by physical, chemical, temperature, or pH stimuli—provides additional functional advantages [81,82].

Despite their benefits, conventional injectable hydrogels face limitations such as gelation timing, biomechanical compatibility, and potential mechanical damage to surrounding tissues, which can compromise long-term function. Recent innovations in shape-memory and self-healing hydrogels address these challenges, enabling restoration of scaffold morphology while preserving cell viability and tissue functionality, thereby paving the way for more effective and durable bone regeneration strategies [82].

### 3.6. Advanced Bioactive Strategies

Beyond the traditional use of growth factors and stem cells, emerging approaches in bone tissue engineering highlight bioactive platforms that utilize advanced delivery and signalling mechanisms to shape the regenerative microenvironment. The development of smart implants represents an innovative approach in which advanced materials science is integrated with biological mechanisms to deliver structural support while simultaneously enhancing osteogenesis, preventing infection, promoting vascularization, modulating immune activity, and enabling neuromodulatory osteogenesis [83]. These functionalities arise from carefully engineered material designs—such as bioactive coatings, surface nanostructures, and other surface modifications—that dynamically interact with the cellular microenvironment to improve clinical outcomes [84]. In recent years efforts are focused on the design of synthetic smart bone implants, along with the refinement of their functions and fabrication strategies, such as the induction and modification of bioactive complexes including exosomes and functional proteins. At the same time, considerable research has been devoted to the regulation of macrophage polarization (M1 and M2 phenotypes) by tailoring implant surface topographies and incorporating drugs, cytokines, and bioactive metal ions. Collectively, these advances underscore the importance of the immunomodulatory role of implants as a fundamental mechanism facilitating osteointegration and bone defect repair [85].

Liposomes and other lipid-based nanomedicines, protein nanoparticles, polymeric micelles and nanoparticles, polymer–drug conjugates, and various inorganic nanoparticles are among the most widely studied candidates proposed for nanomedical applications [86]. Mesoporous silica nanoparticles (MSNs), among the most studied nanocarriers, have attracted significant attention for smart drug delivery owing to their favourable physicochemical characteristics. Various studies highlight the effectiveness of silica-based mesoporous nanomatrices in transporting antitumoral [87,88] and imaging agents [89] to bone cancer cells. By incorporating phosphorus groups, SBA-15 mesoporous silica nanomatrices achieved increased alendronate loading and induced apatite formation, making them highly promising for applications in osteoporosis therapy [90]. Silica-based mesoporous glasses incorporated into PLGA or MSNs embedded in porous collagen–gelatin scaffolds [91] have shown controlled vancomycin release for bone infection treatment. Moreover, MSN-loaded scaffolds allow the concurrent delivery of multiple therapeutics; for example, cephalexin can be confined within the mesopores while vascular endothelial growth factors are integrated into the scaffold matrix, enabling simultaneous bacterial eradication and bone regeneration [92].

In tissue engineering, combining gene-based therapies with scaffolds typically involves either loading the scaffolds with transfection/transduction vectors (gene- and RNAi-activated scaffolds) to induce in situ modification of host cells, or pre-seeding the scaffolds with cells that have been transfected or transduced with the target gene(s) before implantation. To promote the expression of specific proteins, gene-activated scaffolds can be loaded with various agents, including pDNA, viral vectors, and RNA transcripts. Complexing pDNA with cationic polymers, such as PEI (polyethylenimine), forms nanoplexes that are endocytosed by cells, enabling nuclear delivery and gene expression. While the precise mechanisms of decomplexation and nuclear translocation remain unclear, this non-viral approach has proven effective for inducing gene expression [93]. Khorsand et al. loaded PEI-pDNA nanoplexes encoding BMP-2 and/or FGF-2 (Fibroblast Growth Factor-2) onto collagen scaffolds to evaluate whether co-expression of the two growth factors could synergistically enhance bone regeneration. In vitro, co-delivery of BMP-2 and FGF-2 nanoplexes to human bone-marrow-derived mesenchymal stem cells (BMSCs) doubled BMP-2 expression compared to single-gene treatments. In a diabetic rabbit long bone model, scaffolds loaded with both nanoplex formulations yielded greater bone regeneration (133.7 mm^3^ new bone) and higher union rates than scaffolds delivering FGF-2 (96.4 mm^3^) or BMP-2 (82.2 mm^3^) alone [94]. Malek-Khatabi et al. developed a gene-activated scaffold that releases BMP-2–encoding chitosan-pDNA nanoplexes in response to enzymatic activity from infiltrating cells [95]. They modified electrospun PCL mats with MMP (metalloproteinases) -sensitive peptide sequences and loaded them with homogeneous nanoplexes produced via a microfluidic system. The scaffolds demonstrated MMP-triggered nanoplex release (≈60% over 24 h), enhanced osteogenic differentiation of MSCs in vitro, and superior bone regeneration in a rat calvarial defect model (~18 mm^3^ for modified scaffolds vs. ~9 mm^3^ and ~4 mm^3^ for unmodified scaffolds and controls) [95].

RNAi-activated scaffolds deliver siRNAs, shRNAs, or miRNAs to silence specific genes and modulate protein expression through RNA interference. This regulation induces phenotypic changes in cells and alters the local microenvironment, supporting bone regeneration. Zhang et al. demonstrated that incorporating miR-26a nanoplexes encapsulated within slow-release PLGA microspheres outperformed both fast-release platforms and those lacking miR-26a in a mouse ectopic bone formation model (~4.3 mm^3^ vs. ~1.6 mm^3^ and 0 mm^3^ for fast-release and no miR-26a, respectively) and in a 5 mm calvarial defect model (~100% vs. ~39% and ~22% for fast-release and no miR-26a, respectively) [96].

Collectively, these advanced bioactive strategies represent a shift toward multifunctional and responsive biomaterials, designed not only to provide structural support but also to actively guide and accelerate the complex biological processes underlying bone regeneration.

## 4. Conclusions

A systematic review of the current state of bone substitutes in regenerative medicine, based on publications from 2019 to May 2025, has identified key patterns in their biological and mechanical properties that determine their clinical application across various medical specialties. The critical role of porosity in the functionality of synthetic bone substitutes has been established, with structures featuring pore sizes from 23 to 210 micrometres providing optimal osteoconduction, vascularisation, and cellular infiltration, while maintaining mechanical strength at up to 70% open porosity.

The review demonstrates the transformation of bone substitute technologies from traditional structural support materials to bioactive systems with osteoinductive properties. A systematization of the main categories of bone substitutes has shown that autogenous grafts possess the highest osteogenic, osteoinductive, and osteoconductive properties, owing to the complex activity of growth factors, including BMP-2, BMP-7, and concentrated growth factors. However, critical limitations of autografts have been identified, with 20–30% of patients experiencing persistent donor site pain and limited material availability for the reconstruction of significant bone defects, which complicate approximately 6% of all fractures with nonunion. Allogeneic and xenogeneic substitutes demonstrate moderate osteoconductive properties and preserve structural integrity, but require intensive processing to minimize the risks of infection transmission and immune reactions, in accordance with European Directives 2004/23/EC and 2006/86/EC. Synthetic substitutes—based on calcium phosphate ceramics, biodegradable polymers, and bioactive glass—offer controlled degradation and excellent biocompatibility, but lack intrinsic osteogenic potential unless combined with bioactive factors. Advanced bioactive strategies illustrate a transition toward multifunctional, responsive biomaterials that not only provide structural support but also actively modulate the cellular microenvironment to enhance and accelerate bone regeneration. Recent advances in bioactive and composite biomaterials, including hydrogels, energetic and metabolic regulatory materials, demonstrate that integrating bioelectric and bioenergetic cues with tailored scaffold designs can actively guide cell behaviour and metabolism, offering a promising strategy for more effective and durable bone regeneration.

The clinical feasibility of specific types of bone substitutes is determined by the particular requirements of individual medical fields. In orthopedic traumatology, autogenous grafts and synthetic calcium phosphate materials with high mechanical strength are preferred for load-bearing applications. In dentistry and maxillofacial surgery, xenogeneic bovine materials are favoured due to their volumetric stability and reliable implant integration. In neurosurgery, materials with controlled degradation and high biocompatibility are essential, with autogenous dentin matrices and synthetic polymers proving effective. The study confirmed the suitability of β-tricalcium phosphate for long-term applications due to its optimal degradation profile, in contrast to calcium sulphate, which exhibits overly rapid resorption. The highest regeneration rates are observed when synthetic scaffolds are combined with mesenchymal stem cells and extracellular vesicles, which significantly enhance the osteogenic potential of these materials.

The future of bone substitution is likely to be shaped by the integration of advanced biomaterials, bioactive cues, and personalized scaffold designs. Emerging materials, including metabolic regulatory biomaterials, electrically active scaffolds, and self-healing hydrogels, offer the potential to modulate cell behaviour and tissue regeneration more effectively than traditional autogenous, allogenic, or synthetic substitutes. Composite scaffolds combining organic and inorganic components can further enhance mechanical stability while providing biologically active environments that promote osteogenesis and vascularization.

Personalized approaches, particularly 3D bioprinting, will enable the fabrication of patient-specific scaffolds that conform to complex anatomical defects, potentially reducing complications associated with donor site morbidity or immune reactions. In parallel, integrating bioelectric and bioenergetic cues into scaffold design may further accelerate tissue repair by regulating cellular metabolism, redox balance, and signalling pathways. Achieving these advances will require interdisciplinary collaboration between materials scientists, bioengineers, and clinicians, as well as the establishment of standardized regulatory frameworks to ensure safety, efficacy, and clinical translatability. Together, these strategies promise a new generation of bone substitutes that are both functionally superior and clinically practical.

A limitation of the study is the absence of long-term clinical outcome analyses concerning the use of different bone substitutes in patients with various comorbidities. Future research should focus on the development of fully biodegradable composite systems with controlled growth factor release, alongside the integration of 3D bioprinting technologies to create individualized tissue-engineered solutions incorporating triply periodic minimal surfaces.

## Figures and Tables

**Table 1:** All articles included in the present study.

Reference	Aim of the Study	Type of Material	Field of Application	Type of Study	Outcomes of the Bone Substitute
Ferraz, M.P. [1]	To analyze and compare bone grafting materials used in dental and maxillofacial surgery, focusing on their properties, benefits, and clinical application challenges	Autologous bone, allografts, xenografts, synthetic or xenogeneic scaffolds with growth factors to enhance their osteogenic properties	Dentistry, oral and maxillofacial surgeries	In vivo and clinical research data	Osteogenic properties
Heitzer, M. et al. [2]	To investigate the osteogenic potential of dental pulp stem cells (DPSCs) on three types of bone graft substitutes—alloplastic (BCP), allogeneic (FDBA), and xenogeneic (DBBM)—under in vitro conditions	Alloplastic, allogeneic, and xenogeneic bone graft substitutes	Dentistry, oral and maxillofacial surgeries	In vitro	Osteogenic (bone-forming) potential
Fattahian, H. et al. [3]	To review current materials and methods for the repair and regeneration of bone defects, as well as new tissue engineering approaches to improve the treatment of bone injuries	Autografts, allografts, and xenografts	Orthopedics	Systematic reviews	Biocompatibility, osteoconductivity, osteoinductivity, mechanical strength, resorbability, availability, combinability with biofactors
Dahiya, U. et al. [4]	To analyze several bone substitutes, such as synthetics, bioceramics, and polymers, as a substitute for autologous or allogeneic bone for the treatment of bone defects. These bone substitutes should be biocompatible, bioresorbable, osteoconductive, osteoinductive, and promote new bone growth	Synthetics bone substitutes	Orthopedics, dentistry, oral and maxillofacial surgeries	Review	Biocompatibility, bioresorbability, osteoconductivity, osteoinductivity, support of new bone ingrowth
Manzini, B. et al. [5]	To provide an overview of the bone tissue, including the role of stem cells and some of the bioactive molecules associated with these processes	Synthetic bone substitutes, hydroxyapatite (HA), β-tricalcium phosphate (β-TCP), biphasic calcium phosphate (BCP), combinations of HA and TCP	Orthopedics, dentistry, oral and maxillofacial surgeries	Review	Stem cells and bioactive factors are key for regeneration
Miletić, M. et al. [6]	This in vivo study demonstrates for the superior bone regenerative capacity of CAP-pretreated β-TCP seeded with PDLSCs, highlighting the therapeutic potential of this combined approach in osteoregeneration	Beta-Tricalcium Phosphate	Orthopedics, dentistry, oral and maxillofacial surgeries	In vivo	Periodontal ligament stem cells with beta-tricalcium phosphate treated by cold atmospheric plasma significantly enhanced bone regeneration
Mukhlis, S. et al. [7]	To investigate the therapeutic potential of adipose-derived pericytes, in combination with biomaterials and growth factors, for enhancing bone tissue regeneration	Injectable hydrogels and polymer (gelatin, alginate, collagen, chitosan, poly-L-lactic acid, hyaluronic acid, fibrin, heparin, and polyethylene glycol) scaffolds with pericytes	Dentistry, oral and maxillofacial surgeries	In vivo	Preclinical studies show that pericytes combined with scaffolds or DBM enhance bone healing
Dőri, F. et al. [8]	To evaluate the histological findings of retrieved ePTFE membranes using PRP and bone substitutes, the effect of PRP on graft materials, and the correlation of the findings with the clinical outcomes	PRP + Beta-Tricalcium Phosphate + non-resorbable membrane (GTR)	Dentistry, oral and maxillofacial surgeries	In vivo	Application of β-TCP and PRP may enhance membrane integration and periodontal healing
Zhang, Q. et al. [9]	To analyze tissue engineering and regenerative therapies targeting cell senescence in bone and cartilage, with a focus on treating osteoporosis and age-related degenerative conditions through the use of biomaterials, growth factors, and cell-based strategies	Bone grafts with antiosteoporotic growth factors	Orthopedics	Review	Bone grafts combined with osteoporosis therapies—including scaffolds releasing anti-osteoporotic drugs or implants with therapeutic surface modifications—can improve low bone density and enhance impaired bone regeneration
Rupp, M. et al. [10]	To evaluate the overall use of bone graft substitutes, autografts and allografts, of different types of bone graft substitutes (calcium sulphate, calcium phosphate, calcium phosphate ceramics or polymethyl methacrylate) and of different bone grafts (cancellous vs. cortical), and the use of antibiotic-loading of bone graft substitutes in orthopedic surgery in Germany	Autografts and allografts, synthetic bone substitutes (calcium sulphate, calcium phosphate, calcium phosphate ceramics or polymethyl methacrylate)	Orthopedics	In vivo	Increasing use of bone graft substitutes and antibiotic-loaded bone graft substitutes
Laubach, M. et al. [11]	To review and evaluate current methods for the treatment of bone defects, with a particular focus on the potential and challenges of using personalized 3D-printed implants for long bone regeneration	3D-printed scaffolds	Orthopedics	Review	3D-printed scaffolds provide osteoconductive surfaces, mechanical support, and containment for bone grafts, enhancing bone ingrowth
Santoro, A. et al. [12]	To analyze current natural and synthetic bone substitutes used in orthopedic surgery, with a focus on innovations and challenges in the treatment of complex bone defects	Natural grafts—comprising autologous, allogeneic, and xenogeneic materials; synthetic alternatives, including biodegradable and non-biodegradable biomaterials	Regenerative medicine and orthopedic surgery	Review	Natural grafts (autologous, allogeneic, xenogeneic) offer biological advantages, whereas synthetic substitutes (biodegradable and non-biodegradable) provide structural versatility and lower immunogenicity
Tournier, P. et al. [13]	To evaluate the regenerative potential of a novel partially demineralized allogeneic bone paste as an alternative to traditional allogeneic grafts, focusing on enhanced bone healing and user-friendly application in challenging skeletal defect areas	Demineralized allogenic bone graft in the form of a paste	Orthopedic surgery	In vivo	The bone paste supported bone healing in guided bone regeneration and critical-size defect models
Parikh, S. et al. [14]	To present the ethical and clinical approach in a case of triple bone marrow donation from a single pediatric donor to three HLA-identical siblings with the same primary immunodeficiency, aiming to guide future decision-making in similar cases	Allogeneic bone grafts	Regenerative medicine	In vivo	Multiple harvests from pediatric donors can be performed safely with careful clinical and ethical oversight
Sharifi, M. et al. [15]	To explore the sources, advantages, challenges, and techniques of acellularization and recellularization in allogeneic and xenogeneic bone grafts, aiming to advance future bone defect treatment strategies and support product commercialization	Allogeneic and xenogeneic bone grafts	Regenerative medicine	Review	Allogeneic and xenogeneic acellularized bone grafts mimic native bone structure and support osteoconduction and osteoinduction
Ana, I. [16]	To review the biological aspects, advantages, and disadvantages of various bone graft substitutes used in dental implantology, with a focus on calcium phosphate-based materials	Calcium phosphate-based bone substitutes	Dentistry, oral and maxillofacial surgeries	Review	Advantages of calcium phosphate-based bone substitutes in dentistry
Janjua, O.S. et al. [17]	To investigate the potential and applications of autogenous tooth grafts for regenerating maxillary and mandibular bone defects, including sinus and ridge augmentations and socket preservation before implant placement	Autogenous tooth graft	Dentistry, oral and maxillofacial surgeries	Review	Autogenous tooth bone grafts exhibit osteoconductivity and osteoinductivity, similar to natural bone
Malik, S. et al. [18]	To evaluate the efficacy of adjuvant therapy with Concentrated Growth Factor (CGF) along with xenograft and autograft in mandible fractures, in terms of bone density gain and healing over the period of 6 months	Xenograft and autograft with Concentrated Growth Factor	Dentistry, oral and maxillofacial surgeries	In vivo	CGF enhances bone regeneration with both autograft and xenograft
Black, C. et al. [19]	To evaluate the effectiveness of Laponite™ nanoclay gel as a carrier for localized BMP-2 delivery in bone regeneration, using a relevant large animal preclinical model with femoral condyle defects in aged sheep	Synthetics Smectite nanoclay gel (Laponite™) combined with absorbable collagen sponge and BMP-2 (Bone Morphogenetic Protein-2)	Bone tissue regeneration	In vivo and clinical research data	Autograft showed superior bone formation, nanoclay gels exhibited excellent biocompatibility and potential for delivering bone morphogenetic protein-2 locally
Jin, Y. et al. [20]	To investigate the effectiveness of demineralized bone matrix (DBM) as a carrier for Escherichia coli-derived recombinant human BMP-2 (ErhBMP-2) in bone regeneration	Autograft, demineralized bone matrix (DBM) as a bone graft substitute and growth factor carrier	Orthopedic surgery, bone tissue regeneration	In vivo	DBM as the carrier showed significantly higher bone volume and bone thickness than the groups with HA as the carrier
Tasdemir, U. et al. [21]	To investigate the effect of combining autogenous bone graft with recombinant human vascular endothelial growth factor (rhVEGF) on bone regeneration in a rat mandibular defect model	Autogenous bone graft, gelatin sponge plus rhVEGF, autogenous bone graft plus rhVEGF	Dentistry, oral and maxillofacial surgeries	In vivo	Combining autogenous bone grafts with rhVEGF could potentially improve neovascularization and enhance bone regeneration.
Taşdemir, U. et al. [22]	To evaluate all the tooth layers mixed with simvastatin without any demineralization process effect on bone formation	Autogenous tooth bone grafts (ATGM) with simvastatin, xenogenic bone graft	Dentistry, oral and maxillofacial surgeries	In vivo	Autogenous mineralized tooth bone graft should be mixed with simvastatin for bone regeneration
Kunze, K. et al. [23]	To perform a meta-analysis of RCTs evaluating donor site morbidity after bone-patellar tendon-bone (BTB), hamstring tendon (HT) and quadriceps tendon (QT) autograft harvest for anterior cruciate ligament reconstruction (ACLR)	BTB autograft	Orthopedic surgery	Systematic reviews	Autograft selection should be personalized through considering differential rates of donor-site morbidity
Attia, A. et al. [24]	To evaluate the safety and donor site morbidity of autologous cancellous bone grafts harvested from the distal lower extremity (calcaneus, proximal, and distal tibia) through a meta-analysis of published research	Autologous cancellous bone grafts	Orthopedic surgery	Systematic reviews	Calcaneal, distal tibial, and proximal tibial bone autografts are safe with a low rate of overall and major complications
Khalid, M. et al. [25]	To assess the donor site morbidity in patients having anterior cruciate ligament reconstruction (ACLR) using peroneus longus tendon (PLT) autograft	Autograft	Orthopedic surgery	In vivo	ACLR using the PLT autograft resulted in a good functional outcome, smooth rehabilitation with an early return to sports, and minimal complications at the donor site
Sun, H. et al. [26]	This paper comprehensively explores the composition, mechanisms underlying osteoinductivity, preparation methods, and clinical applications of ADM with the aim of establishing a fundamental reference for future studies on this subject	Autogenous bone	Dentistry, oral and maxillofacial surgeries	Review	ADM has a low rejection rate, possesses osteoinductive properties, and is characterized by easy preparation
Liu, J. et al. [27]	To explore the potential effect of three allogenic bone substitute configurations on the viability, adhesion, and spreading of osteoblasts in vitro	Allogenic bone substitute	Orthopedic	In vitro	Bone powder and bone granule promote cell adhesion and spreading compared to bone fibre group
European Parliament and Council [28]	Setting standards of quality and safety for the donation, procurement, testing, processing, preservation, storage and distribution of human tissues and cells	Allogeneic bone tissue	Orthopedics, dentistry, oral and maxillofacial surgeries, neurosurgery	In vivo	Not applicapable
European Commission [29]	To lay down technical requirements for the coding, processing, preservation, storage and distribution of human tissues and cells	Allogeneic bone tissue	Orthopedics, dentistry, oral and maxillofacial surgeries, neurosurgery	In vivo	Not applicapable
Ilyas, I. et al. [30]	To present the 10-year experience of the bone bank at KFSH&RC, detailing its structure, donor/recipient protocols, graft processing and safety systems, and the clinical use of allografts in surgeries such as joint revision, spine, and tumour procedures	Allografts	Orthopedics, neurosurgery	Clinical, preclinical	Administrative structure, donor and recipient testing protocols, allograft retrieval, processing procedures, and safety arrangements
Regmi, A. et al. [31]	To present the establishment and management of an orthopedic bone bank in India, focusing on the legal, medical, and organizational aspects of using allogenic bone for treating bone defects	Allografts	Orthopedics	Review	Allogenic bone grafts, supported by bone banking systems, provide a safe alternative to autografts, though they require strict legal, medical, and organizational regulation
Asamoto, T. et al. [32]	To analyze the 15-year trends in the supply and use of bone allografts in a regional bone bank in Japan and to assess the effectiveness of the donor screening process	Bone allografts	Orthopedics, dentistry, oral and maxillofacial surgeries, neurosurgery	Preclinical	The study highlights the importance of thorough screening processes for allograft donors to minimize the number of discarded grafts
American Association of Tissue Banks. AATB Standards Rebuild [33]	These regulations and practices underpin modern bone grafting procedures, aiming to optimize clinical outcomes while minimizing associated risks	Allografts	Orthopedics, dentistry, oral and maxillofacial surgeries, neurosurgery	Preclinical	Standards for Tissue Banking
Um, I. et al. [34]	To summarize preclinical studies on the osteoinductive potential and antigenicity of allogeneic demineralized dentin matrix (Allo-DDM), in order to evaluate its viability as a reliable bone graft substitute, particularly at extraskeletal sites	Allogeneic demineralized dentin matrix	Dentistry, oral and maxillofacial surgeries	Review	Allo-DDM showed great potential for osteoinductivity in extraskeletal sites with low antigenicity
Waletzko-Hellwig, J. et al. [35]	To evaluate high hydrostatic pressure (HHP) as a gentle sterilization method for allogenic bone substitutes by assessing its effects on the mechanical properties of treated bone granules and trabecular bone, in comparison to untreated samples	Allogenic bone substitutes	Orthopedics	Preclinical	HHP treatment is suitable alternative to current processing techniques for allogenic bone substitutes since with no negative effects on mechanical properties occurred
Shao, A. et al. [36]	To investigate whether GGTA1/iGb3S double knockout (G/i DKO) mice are sensitive to Gal antigen-positive xenoimplants and assess their suitability as a model for studying α-Gal-mediated immunogenicity in xenotransplantation	Xenogeneic bone grafts	Orthopedics, dentistry, oral and maxillofacial surgeries, neurosurgery	Preclinical	Evaluating the α-Gal-mediated immunogenic risk of xenogeneic grafts
Amid, R. et al. [37]	To systematically review and evaluate the structural and chemical properties of various xenograft bone substitutes based on in vitro studies	Xenogeneic bone grafts	Orthopedics, dentistry, oral and maxillofacial surgeries, neurosurgery	Systematic reviews	Xenograft bone substitutes, mainly bovine-derived, show structural similarity and biocompatibility with human bone, but their properties (porosity, crystallinity, Ca/P ratio, and osteogenesis) vary significantly depending on preparation methods. Proper pore size and interconnectivity are crucial for effective bone regeneration
Jerbić Radetić, A.T. et al. [38]	To investigate the biological properties of a new bovine xenogeneic biomaterial enriched with magnesium alloy in a 5 mm critical-sized bone defect (CSBD) model, and to compare its osteoconductive potential with existing xenogeneic biomaterials (Cerabone^®^, Cerabone^®^ + autologous bone, and OsteoBiol^®^) for possible use in oral implantology	Xenogeneic bone grafts	Dentistry, oral and maxillofacial surgeries	Preclinical, in vivo	Novel bovine xenogeneic biomaterial enriched with magnesium alloy in a 5 mm CSBD model, compared to Cerabone^®^ and OsteoBiol^®^, the Cerabone^®^ + Mg group showed higher bone volume, faster biodegradation, and strong osteoinductive properties
Pröhl, A. et al. [39]	To evaluate the inflammatory tissue response and bone healing capacity of a newly developed xenogeneic bone substitute material (BSM) combined with hyaluronate (HY), compared to a control BSM without HY and a sham operation group, using a rat calvaria model over 2, 8, and 16 weeks	Xenogeneic bone substitute	Orthopedics, dentistry, oral and maxillofacial surgeries, neurosurgery	Preclinical, clinical	Both materials proved biocompatible and supported gradual osteoconductive bone regeneration
Denner, J. [40]	To argue that although porcine cytomegalovirus (PCMV) is an important safety concern in xenotransplantation, there are effective methods for its elimination, and therefore, it does not represent an insurmountable obstacle to the advancement of this medical technology	Xenogeneic bone substitute	Orthopedics, dentistry, oral and maxillofacial surgeries, neurosurgery	Preclinical, clinical	To apply methods for eliminating viruses in xenotransplantations
Groenendaal, H. et al. [41]	To develop a risk-based framework for identifying and evaluating porcine microorganisms and parasites (MP) that could pose health risks in xenotransplantation.	Xenogeneic bone substitute	Orthopedics, dentistry, oral and maxillofacial surgeries, neurosurgery	Preclinical, clinical	Provides a structured way to categorize and mitigate the risks posed by porcine microorganisms in xenotransplantation, highlighting that most risks can be addressed with strict biosecurity and monitoring
U.S. Food and Drug Administration [42]	To provide guidance to the industry on the safe development and use of xenotransplantation products in humans	Xenotransplantation products in humans	Orthopedics, dentistry, oral and maxillofacial surgeries, neurosurgery	Preclinical	Guidelines
European Medicines Agency [43]	To provide scientific and regulatory guidance on the quality, safety, and efficacy requirements for xenogeneic cell-based medicinal products intended for human use	Xenogeneic cell-based medicinal products	Orthopedics, dentistry, oral and maxillofacial surgeries, neurosurgery	Preclinical	Guidelines
Hawthorne, W.J. et al. [44]	To support the safe and ethically responsible development of xenotransplantation by promoting international cooperation and coordination	Xenografts	Orthopedics, dentistry, oral and maxillofacial surgeries, neurosurgery	Preclinical	Confirmed the significant progress and the need for global collaboration between science, technology and regulations in the field of xenotransplantation to harmonize practices and rules in different countries
Sánchez-Labrador, L. et al. [45]	To evaluate the feasibility of xenogeneic bone blocks for ridge augmentation compared with autogenous blocks by analyzing block survival rates, block resorption, subsequent implant survival rate, post-surgical complications, and histomorphometric findings	Xenogeneic bone blocks, autogenous blocks	Orthopedics, dentistry, oral and maxillofacial surgeries, neurosurgery	Systematic reviews	Histological and histomorphometric analysis observed more bone formation and less residual bone substitute with autogenous bone blocks than xenogeneic bone blocks
Maevskaia, E. et al. [46]	To evaluate a new design of synthetic bone substitutes based on the ADMS algorithm, which provides high mechanical stability and good osteoconductivity, comparable to traditional lattice structures, and is suitable for clinical use in treating bone defects	Synthetic bone substitutes	Orthopedics, dentistry, oral and maxillofacial surgeries, neurosurgery	Preclinical, in vivo	The study found these substitutes, made from hydroxyapatite (a bone mineral), are highly osteoconductive, meaning they effectively promote the growth of new bone
Ding, Y. et al. [47]	To analyze the advantages and performance requirements of 3D-printed bone scaffolds compared to traditional ones. It reviews key 3D printing technologies used for bone scaffold fabrication, highlighting their benefits and limitations. Although 3D printing shows promise in improving recovery and scaffold performance, material-related challenges remain, and future research directions are suggested	3D-printed bone scaffolds	Orthopedics	Clinical	3D-printed bone scaffolds show clear advantages over traditional ones, offering better performance and faster recovery, though material issues remain
Li, X. et al. [48]	This paper reviews current optical 3D printing methods for tissue engineering scaffold fabrication, highlighting techniques like stereolithography, two-photon polymerization, and others	3D-printed bone scaffolds	Orthopedics, dentistry, oral and maxillofacial surgeries, neurosurgery	Preclinical	Each technique—extrusion-based printing, selective laser sintering, stereolithography, and two-photon polymerization—offers distinct advantages and limitations regarding precision, fabrication speed, material compatibility, and application suitability
Nifant’ev, I. et al. [49]	The study develops bone substitute composites using biodegradable polyesters (PLLA, PCL) and carbonated hydroxyapatite. It shows that adding special compatibilizers improves the composites’ strength, thermal stability, and overall performance, making them suitable for bone surgery and orthopedics	Synthetic bone substitutes	Bone surgery and orthopedics	Preclinical	PLLA composites with 25 wt% pCAp exhibited the best mechanical performance and thermal stability
Kang, T.Y. et al. [50]	This study enhanced the mechanical strength of bioactive glass (BAG) by incorporating varying amounts of zirconia (ZrO_2_) while preserving its bioactive properties. ZrO_2_-BAG showed improved toughness, slower degradation, good biocompatibility, and promoted cell growth and bone formation. In vivo tests confirmed its potential as a strong and effective bone graft substitute	Synthetic bone graft substitute	Bone surgery and orthopedics	Preclinical, In vivo	Incorporation of zirconium oxide (ZrO_2_) into bioactive glasses improved their mechanical strength while preserving porosity
Ferraz, M.P. et al. [51]	This review focuses on methods to produce nanophased HA through precipitation and discusses both traditional and recent advances in HA nanoparticle applications	Synthetic bone graft	Orthopedics, dentistry, oral and maxillofacial surgeries, neurosurgery	Review	Nanophased HA obtained via precipitation methods shows strong promise for improving the bioactivity and functionality of synthetic ceramics
Cheng, L. et al. [52]	This article reviews recent advances in 3D printing for bone repair, highlighting its ability to create custom scaffolds using biomaterials and living cells. It covers key printing techniques (SLA, SLS, FDM, inkjet) and their clinical potential in regenerating large bone defects	Synthetic 3D printing for bone repair	Orthopedics, dentistry, oral and maxillofacial surgeries, neurosurgery	Review	Various biomaterials and nanomaterials have been successfully applied in 3D-printed scaffolds, while major printing methods—including SLA, SLS, FDM, and ink-jet printing—have been utilized to create constructs with structural and functional similarities to bone tissue
Liang, D. et al. [53]	To present a simple method for fabricating porous tantalum (Ta) with controlled pore sizes and bone-like mechanical properties, demonstrating its excellent biocompatibility and effectiveness in supporting bone repair in vitro and in vivo	Synthetic bone graft	Orthopedics, dentistry, oral and maxillofacial surgeries, neurosurgery	In vitro, in vivo	The porous tantalum demonstrated excellent biocompatibility in vitro and effective bone repair in vivo in a rat femur defect model, validating it as a viable bone substitute
Kumar, A. et al. [54]	To developed biodegradable bone scaffolds using a blend of polycaprolactone (PCL)	Synthetic bone graft	Orthopedics, entistry, oral and maxillofacial surgeries, neurosurgery	Preclinic	Increasing PLGA content from 25 wt.% to 75 wt.% accelerated the biodegradation rate threefold within 2 weeks in phosphate-buffered saline
Celik, D. et al. [55]	To developed a hybrid hydroxyapatite (HA) scaffold mimicking both cortical and cancellous bone using slip casting and freeze-drying	Synthetic bone graft	Orthopedics, dentistry, oral and maxillofacial surgeries, neurosurgery	Preclinic	Hybrid scaffold demonstrated 70% open porosity, adequate mechanical strength, and structural resemblance to natural bone. Analyses confirmed its suitability for supporting vascularization, osteoinduction, and osteoconduction
Marongiu, G. et al. [56]	To evaluate the effectiveness of cements, bone substitutes, and other augmentation techniques in the surgical treatment of proximal humeral fractures	Synthetic bone graft	Orthopedics, dentistry, oral and maxillofacial surgeries, neurosurgery	Systematic review	Calcium phosphate and calcium sulphate injectable composites provided good biocompatibility, osteoconductivity, and lower mechanical failure rate when compared to non-augmented fractures
Kang, H. et al. [57]	To fabricate and compare multichannel biphasic calcium phosphate (BCP) and β-tricalcium phosphate (TCP) scaffolds in terms of their biodegradation and bone regeneration capabilities over time	Synthetic bone graft- biphasic calcium phosphate, β-tricalcium phosphate	Orthopedics, dentistry, oral and maxillofacial surgeries, neurosurgery	Preclinic	BCP exhibited higher compressive strength, while TCP showed greater calcium and phosphorus ion release in SBF. Both scaffolds demonstrated excellent in vitro biocompatibility
Kowalewicz, K. et al. [58]	To evaluate the in vivo degradation, osseointegration, and biocompatibility of 3D powder-printed calcium magnesium phosphate cement (CMPC) scaffolds (Mg225 and Mg225d) compared to tricalcium phosphate (TCP), assessing their potential as bone substitutes	Synthetic bone graft	Orthopedics, dentistry, oral and maxillofacial surgeries, neurosurgery	In vivo	All scaffolds showed excellent biocompatibility and gradual osseointegration over 6 weeks
Heitzer, M. et al. [59]	To evaluate the osteogenic potential of dental pulp stem cells (DPSCs) and their extracellular vesicles (EVs) on different types of bone grafts (alloplastic, allogeneic, and xenogeneic), and to determine if EVs enhance DPSC proliferation and differentiation for potential use in bone regeneration therapies	Alloplastic, allogeneic, and xenogeneic bone grafts	Dentistry, oral and maxillofacial surgeries, neurosurgery	In vitro	Addition of EVs significantly enhances the biocompatibility of human pulp stem cells with xenogeneic bone grafts, leading to superior cell proliferation, attachment, and osteogenic differentiation compared to other bone grafts tested
Sallent, I. et al. [60]	To highlight successful clinical cases and key factors in the translation of innovative bone substitutes—such as biological glues, stem cell-seeded scaffolds, and gene-functionalized grafts—from research to clinical use, and to inform future strategies for effective bone graft development	Synthetic bone graft- biological glues, stem cell-seeded scaffolds, gene-functionalized grafts	Dentistry, oral and maxillofacial surgeries, neurosurgery	Review	Highlights important ideas and aspects in the field of bone grafting
Massari, L. et al. [61]	To evaluate the safety of porous hydroxyapatite bone substitutes in trauma orthopedic surgery by analyzing adverse events in a retrospective cohort of 114 patients	Synthetic bone graft- porous hydroxyapatite bone substitutes	Orthopedics	Clinical	Porous hydroxyapatite bone substitutes appear safe for use in trauma orthopedic surgery when applied following proper biomechanical principles
Mahardawi, B. et al. [62]	To evaluate the available literature on the Allogenic Demineralized Dentin Matrix (Allo-DDM), revealing its clinical performance when used for implant place-ment procedures	Allogenic demineralized dentin matrix graft	Dentistry, oral and maxillofacial surgeries	Systematic review	Allo-DDM could be a possible alternative to other grafting materials used for bone augmentation and implant placement
Batra, C. et al. [63]	To evaluate the clinical effectiveness of combining recombinant human platelet-derived growth factor-BB (rhPDGF-BB) with xenogeneic bone substitutes in treating severe intrabony periodontal defects	Xenogeneic bone substitutes with platelet-derived growth factor-BB	Dentistry, oral and maxillofacial surgeries	Clinical	Combination of rhPDGF-BB and xenogeneic bone substitutes to be a safe and effective graft for severe periodontal intrabony defects
Zhang, S. et al. [64]	To highlight the potential of autogenous odontogenic materials as a novel and biocompatible bone substitute for jaw restoration, comparing their composition and osteogenic properties with other conventional bone grafts	Autogenous odontogenic materials	Dentistry, oral and maxillofacial surgeries	Review	Autogenous odontogenic materials represent a novel and promising class of bone substitutes, combining scaffold functionality with intrinsic osteogenic stimulation
Jung, K. et al. [65]	To demnstrate the clinical applicability and effectiveness of multichannel granular bone substitutes in treating various orthopedic conditions, including bone tumours, fractures, and defects associated with arthroplasty	Synthetic bone substitutes	Orthopedics	Clinical, in vivo	Bone substitute stabilized the bone defect without any complications, and the defect regenerated slowly during the postoperative period
Baldwin, P. et al. [66]	To review and compare the properties, benefits, and clinical applications of autografts, allografts, and synthetic bone graft substitutes in orthopedic trauma surgery, aiming to guide treatment decisions for bone grafting	Autografts, allografts, and synthetic bone graft substitutes	Orthopedics	Review	Autograft, allograft, and bone graft substitutes all possess their own varying degrees of osteogenic, osteoconductive, and osteoinductive properties that make them better suited for different procedures
Meesuk L. et al. [67]	To evaluate the proliferation and osteogenic differentiation potential of bone marrow and umbilical cord mesenchymal stem cells cultured on 3D-printed hydroxyapatite scaffolds, with or without biomimetic calcium phosphate coating, for applications in bone tissue engineering	Synthetic bone substitutes-3D-printed hydroxyapatite scaffolds, with or without biomimetic calcium phosphate coating	Orthopedics, dentistry, oral and maxillofacial surgeries	Preclinic	Osteogenic differentiation: Cultivation on HA scaffolds resulted in increased alkaline phosphatase ALP activity and increased expression of osteogenic genes and proteins compared to control 2D culture cells
Adamska, P. et al. [68]	To review the literature on teeth autotransplantation, supported by a case report involving the autotransplantation of a third mandibular molar into the site of an extracted infraoccluded first mandibular molar, as well as the utilization of advanced platelet-rich fibrin (A-PRF) alongside autogenous dentin grafts for bone tissue regeneration	Teeth autotransplantation	Dentistry, oral and maxillofacial surgeries	Systematic review	Evaluate the efficacy of autotransplantation, the application of growth factors, and the integration of autogenous dentin grafts in remedying dental deficiencies resulting from reinclusion

**Table 2:** Comparative Characteristics of Xenogeneic Bone Substitutes by Source of Origin.

Source of Origin	Structural Features	Clinical Benefits	Main Disadvantages	Indications For Use
Bull	Mineral structure is as similar as possible to human bone; high crystallinity of hydroxyapatite; preserved trabecular architecture	Excellent osteoconductivity; long-term structural support; predictable resorption (12–24 months); broad clinical evidence base	Slow resorption may delay natural remodelling; potential risk of prion disease transmission (theoretical); religious restrictions for some patients	Dental implantology; sinus lift; alveolar ridge augmentation; filling of small bone defects
Pig	Lower density compared to bovine; higher porosity (60–70%); increased resorption capacity	Better remodelling properties; faster integration with native bone (6–12 months); optimal for young patients with active metabolism	Lower mechanical strength; risk of transmission of PERVs and PCMV; cultural and religious restrictions (Islam, Judaism); higher immunogenicity	Periodontal regeneration; filling of defects after tooth extraction; pediatric maxillofacial surgery
Horse	Intermediate characteristics between bovine and porcine; moderate density; balanced porosity	Alternative for patients with religious restrictions; moderate resorption rate (9–18 months); good biocompatibility after processing	Limited market availability; fewer clinical studies; higher cost due to lower prevalence; potential allergic reactions	Orthopedic surgery (limited use); alternative in case of intolerance to other xenografts

Source: created by the author based on R. Amid et al. [37], A.T. Jerbić Radetić et al. [38], A. Pröhl et al. [39].

**Table 3:** Comparative Characteristics of the Main Types of Bone Substitutes.

Substitute Type	Source	Osteogenicity	Osteoinductivity	Osteoconductivity	Biocompatibility	Main Advantages	Main Disadvantages
Autogenous	Patient’s own tissues	High	High	High	Missing	No rejection, presence of living cells, natural growth factors	Limited availability, donor site morbidity, additional pain
Allogeneic	Human donors	Missing	Limited	Moderate	Good after processing	Large volumes, no donor site morbidity	Risk of infection transmission, need for treatment, slow integration
Xenogenic	Animal tissues	Missing	Low	Moderate	Good after processing	Unlimited availability, structural similarity to human bone	Zoonoses risk, need for careful processing, slow remodelling
Synthetic	Artificial materials	Missing	Missing/Low	Good	Excellent	Controlled properties, no biological risks, 3D printing capabilities	Lack of biological activity, need for additional growth factors

Source: created by the author based on M. Heitzer et al. [2]; M.P. Ferraz et al. [51].

**Table 4:** Comparative Characteristics of Biomechanical Properties of Synthetic Bone Substitutes.

Material Type	Porosity (%)	Compressive Strength (MPa)	Elastic Modulus (GPa)	Degradation Rate	Clinical Application
β-tricalcium phosphate (β-TCP)	60–80	2–12	0.5–3.5	Moderate (6–24 months)	Filling defects carrying moderate loads
Calcium sulphate	30–50	10–30	1–6	Fast (4–8 weeks)	Short-term applications, drug delivery
Hydroxyapatite	55–75	20–80	7–13	Very slow (>24 months)	Long-term structural support, spinal surgery
Bioactive glass with ZrO_2_	40–60	30–140	35–45	Supervised (3–12 months)	Load-bearing areas, dental implantology
3D printed frames with TPMS	65–85	5–30	0.3–2.5	Supervised (6–18 months)	Tissue engineering, individual implants

Source: created by the author based on G. Marongiu et al. [56]; L. Cheng et al. [52]; T.Y. Kang et al. [50].

**Table 5:** Clinical Use of Bone Substitutes by Medical Specialty.

Medical Specialty	Main Indications	Recommended Types of Substitutes	Specific Requirements	Clinical Results
Orthopedic traumatology	Fractures, non-union, large defects	Autogenous, allogenic, synthetic (Ca-phosphate, Ca-sulphate)	High strength, osteoinduction, no immune reaction	High union rates (94% success rate), 6% of fractures are complicated by non-union out of 1,090,167 procedures; donor site morbidity 20–30% with autografts
Spinal surgery	Spondylodesis, segmental stabilization	Allogeneic, synthetic (β-TCP, BMP-2)	Structural support, stimulation of fusion	High fusion rates (85–95% when using BMP-2), β-TCP demonstrates optimal degradation profiles over 6–12 months
Oncological surgery	Tumour resection, restoration of integrity	Combined with growth factors, allogeneic materials	Preservation of anatomy and limb function	Effective restoration of functionality in 9–12 months, multi-channel cylindrical granular substitutes have shown successful regeneration
Maxillofacial surgery and dentistry	Augmentation, sinus lift, implantation	Xenogenic (bovine), Ca-phosphate synthetic, allogenic	Biocompatibility, volumetric stability, implant compatibility	Excellent implantation success rate (92–96%) with autogenous dentin matrices; improved soft tissue healing when combined with PRF
Neurosurgery	Cranioplasty, post-tumour defects	Autogenous dentin, synthetic polymers	Biocompatibility with nervous tissue, controlled degradation	High osteoconductive potential of autogenous dentin (85–90% integration in 6 months); 70% open porosity provides high strength
Revision surgery	Bone restoration, implant integration	Porous hydroxyapatite, combined synthetics	High biocompatibility, long-term stability	Study of 114 patients notes 100% safety with no side effects when using porous hydroxyapatite

Source: created by the author based on I. Ana [16]; A. Santoro et al. [12]; P. Baldwin et al. [66].

**Table 6:** Comparative Overview of 3D Bioprinting Modalities.

Feature	Inkjet Bioprinting	Extrusion-Based Bioprinting	Pressure-Assisted Bioprinting	Laser-Assisted Bioprinting
Mechanism	Droplet ejection via thermal, electrostatic, or piezoelectric forces	Continuous extrusion (SSE/FDM)	Continuous extrusion under pneumatic, plunger, or screw pressure	Laser-induced droplet transfer
Materials	Low-viscosity hydrogels: alginate, gelatin, collagen, chitosan, PEG	Natural (alginate, gelatin, collagen, nanocellulose) and synthetic (PCL, PVA), often blended	Hydrogels, collagen, chitosan, ceramics, PEG, PLA, PCL	Natural polymers (collagen, gelatin, alginate, fibrin), PEG, dECM, nHA, cell-laden bioinks
Cell Compatibility	High	Moderate	High	High
Resolution	High	Moderate	Moderate	Very high
Viscosity Range	Low	High	High	Moderate
Advantages	Low cost, high-throughput, non-contact	High-viscosity bioinks, mechanically robust, multi-material	Cell-friendly, customizable geometry, high-viscosity	Non-contact, precise, complex architectures, high spatial resolution
Limitations	Limited viscosity, low cell density tolerance	Shear stress, lower resolution, slower	Shear stress, bioink optimization required	Equipment cost, thermal stress, viscosity constraints
Applications	Soft tissue models, high-throughput screening	Soft tissue, bone scaffolds, implants	Tissue constructs, high-viscosity bioinks	Complex tissue constructs, bone and vascular models

## Abbreviations

The following abbreviations are used in this manuscript:

MeSH	Medical Subject Headings
BMP	Bone Morphogenetic Proteins
CGF	Concentrated Growth Factor
BMP-2	Bone Morphogenetic Protein-2
BMP-7	Bone Morphogenetic Protein-7
DBM	Demineralised Bone Matrix
rhVEGF	Recombinant Human Vascular Endothelial Growth Factor
ACL	Anterior Cruciate Ligament
GBR	Guided Bone Regeneration
PRF	Platelet-Rich Fibrin
PRP	Platelet-rich plasma
NAAT	Nucleic Acid Amplification Tests
AATB	American Association of Tissue Banks
Allo-DDM	Allogeneic Demineralised Dentin Matrix
HHP	High Hydrostatic Pressure
PCMV/PRV	Porcine Cytomegalovirus/Porcine Retrovirus
PERVs	Porcine Endogenous Retroviruses
β-TCP	β-Tricalcium Phosphate
TPMS	Triply Periodic Minimal Surfaces
PLA	Polylactic Acid
PCL	Polycaprolactone
ZrO_2_	Zirconium Oxide
micro-CT	Micro-Computed Tomography
PLGA	Poly(lactic-glycolic acid)
BCP	Biphasic Calcium Phosphate
DPSCs	Human Dental Pulp Stem Cells
EVs	Extracellular Vesicles
rhPDGF-BB	Recombinant Human Platelet-Derived Growth Factor-BB
CPCs	Calcium phosphate cements
PEG	Polyethylene glycol
ECM	Extracellular matrix
RMAT	Regenerative Medicine Advanced Therapy
